# A Systematic Review of Studies on Genotoxicity and Related Biomarkers in Populations Exposed to Pesticides in Mexico

**DOI:** 10.3390/toxics9110272

**Published:** 2021-10-21

**Authors:** Juana Sánchez-Alarcón, Mirta Milić, Vilena Kašuba, María Guadalupe Tenorio-Arvide, José Mariano Rigoberto Montiel-González, Stefano Bonassi, Rafael Valencia-Quintana

**Affiliations:** 1 Laboratorio “Rafael Villalobos-Pietrini” de Toxicología Genómica y Química Ambiental, Facultad de Agrobiología, Universidad Autónoma de Tlaxcala, CA Genética y Ambiente UATLX-CA 223, Red Temática de Toxicología de Plaguicidas, Tlaxcala 90120, Mexico; xcaretchava@hotmail.com (J.S.-A.); leomonti26@hotmail.com (J.M.R.M.-G.); 2 Mutagenesis Unit, Institute for Medical Research and Occupational Health, 10000 Zagreb, Croatia; vkasuba@imi.hr; 3 Departamento de Investigación en Ciencias Agrícolas, Benemérita Universidad Autónoma de Puebla, Puebla 72570, Mexico; tenorio.arvide@correo.buap.mx; 4 Department of Human Sciences and Quality of Life Promotion, San Raffaele University, 00166 Rome, Italy; stefano.bonassi@sanraffaele.it; 5 Unit of Clinical and Molecular Epidemiology, IRCCS San Raffaele Pisana, 00166 Rome, Italy

**Keywords:** comet assay, DNA damage, micronucleus assay, nuclear abnormalities, sisters’ chromatid exchange

## Abstract

In agricultural activities, pest control is essential, and the most effective method is the use of chemical agents that also represent an important source of exposure to potentially toxic compounds. Pesticides constitute a heterogeneous group of compounds designed specifically to control different pests. Besides measuring their levels or that of their metabolites in air, plasma, serum, blood, urine, etc., some studies reported increased DNA damage levels after occupational or environmental pesticides exposure, evidenced by several cytogenetic biomarkers such as chromosomal aberrations (CA), sister chromatid exchanges (SCE), micronuclei frequency (MN) together with other nuclear abnormalities (NA), alkaline comet assay, but also changes in oxidative stress parameters and miRNA levels. Single or combined, these techniques have also been used in genotoxic biomonitoring studies of workers occupationally exposed to pesticides in Mexico. Despite being a country with great agricultural activity and reported excessive pesticide use, genotoxic studies have been relatively few and, in some cases, contradictory. A review was made of the studies available (published until the end of 2020 on PubMed, Web of Science, Redalyc and Scielo, both in English and Spanish) in the scientific literature that evaluated occupational exposure of human samples to pesticides assessed with DNA damage and related biomarkers in Mexico.

## 1. Introduction

Pesticide application remains the most effective and accepted practice for crop protection, contributing significantly to the increase of agricultural productivity [1]. Due to their persistence, pesticides are ubiquitous pollutants of our environment and have been found in air, soil, water, as well as in human and animal tissues [2]. They differ greatly in their mode of action, form, metabolism, elimination, and toxicity in the organism. Although some of them are not harmless, their use can lead to adverse effects, in the medium and long terms, which is a danger not only to the environment but also to the human population exposed to them or their products from physical and/or biological degradation [1]. Many pesticides can induce genetic damage, in addition to producing various adverse effects on health, affecting the immune, nervous, endocrine, and reproductive systems [3,4,5]. The International Agency for Research on Cancer (IARC) classified a wide range of these compounds as carcinogenic [3,6], among them 56 as carcinogenic in laboratory animals [6], with some associated with cancer in humans [1,7] including cervical and breast cancer [8,9,10,11,12,13,14,15,16]. Although the IARC made its pesticide classification in 2014 [6], the Advisory Group for Monograph Priorities (2015–2019) is still dealing with the risk assessment and safe handling of many pesticide groups, and a final report has not been published yet.

The United States Environmental Protection Agency (USEPA) launched in 2017 the Agricultural Worker Protection Standard for better protection and handling of pesticides and its residues [17]. The European Food Safety Authority (EFSA) published in 2017 a new detailed analysis estimation performed on the pesticide occurrence data in relevant food products consumed and the dietary risk related to the EU consumers’ exposure to pesticide residues [18]. The Food and Agriculture Organization of the United Nations (FAO) and World Health Organization (WHO) made an expert working group (JMPS) that gives recommendations on the adoption, extension, modification, or withdrawal of specifications and develops guidelines and procedures with relevance to the registration and quality control of pesticides in national or regional authorities [19,20].

The population is inevitably exposed to environmental pollution or occupational use. While the information on their acute toxicity is extensive, knowledge about the delayed effects is much more limited. Occupational contact occurs at all stages of pesticide development, formulation, manufacture, and application, which involves exposure to complex mixtures of different active ingredients, “inert” components and by-products present in commercial formulas such as impurities, solvents and other compounds, in addition, the “inert” ingredients, although they do not have pesticide activity, can be biologically active and on some occasions be more toxic for exposed organisms [1]. In 1992, the WHO reported three million cases of intoxication by pesticides and 220,000 of deaths annually registered worldwide [21]. However, in Mexico, despite being a country with great agricultural activity and excessive use of this type of compound, toxicological studies have been relatively few. With a lack of specific records, in 2016, intoxications and poisonings in Mexico caused 1400 death (87% adults and 13% children), and the third cause (13.9%) of intoxication was by pesticide exposure [22], with agricultural and industrial workers as the most affected [23], but also with the general population using contaminated sinkholes, through air spraying, contaminated groundwater, household dust, well using and contaminated tap water [24,25]. In the few monitoring studies in Mexico, there was pesticide bioaccumulation in mothers’ breast milk and adipose tissue [26,27], with higher concentrations of pesticides found in blood, plasma and urine in occupationally exposed workers, than in non-exposed controls, but also higher concentrations of pesticides or their metabolites or their effect were found in children and pregnant women, as vulnerable population groups [28,29,30]. There was also a connection found between pesticide exposures and different types of cancer [7]. Biomonitoring studies focused on genomic modifications have been carried out in populations exposed to pesticides in different countries to determine the risk associated with exposure [1]. However, in Mexico, these types of studies have been relatively few. Therefore, the purpose of this work was to review recent studies in Mexico that evaluated potential genetic damage in individuals occupationally exposed to pesticides.

## 2. Materials and Methods

The PubMed and Web of Science database (Indexed as also SCI-EXPANDED, SSCI, A&HCI, CPCI-S, CPCI-SSH, BKCI-S, BKCI-SSH, ESCI, CCR-EXPANDED, IC.) were searched for all the years with the last date of search on 20 November 2020. The articles were searched both in English and in Spanish.

PubMed was searched with these terms: “mexico” [MeSH Terms] OR “mexico” [All Fields] OR “mexico s” [All Fields] OR “mexico” [All Fields]) AND (“pesticidal” [All Fields] OR “pesticide s” [All Fields] OR “pesticides” [Pharmacological Action] OR “pesticides” [MeSH Terms] OR “pesticides” [All Fields] OR “pesticide” [All Fields]).

For DNA damage: AND (“dna damage” [MeSH Terms] OR (“dna” [All Fields] AND “damage” [All Fields]) OR “dna damage” [All Fields]).

For nuclear anomalies: AND ((“nuclear” [All Fields] OR “nuclears” [All Fields]) AND (“abnormalities” [MeSH Subheading] OR “abnormalities” [All Fields] OR “anomalies” [All Fields] OR “anomalie” [All Fields] OR “anomaly” [All Fields])).

For miRNA: AND (“micrornas” [MeSH Terms] OR “micrornas” [All Fields] OR “mirna” [All Fields] OR “mirnas” [All Fields] OR “mirna s” [All Fields]).

For sister chromatid exchange: AND (“sister chromatid exchange” [MeSH Terms] OR (“sister” [All Fields] AND “chromatid” [All Fields] AND “exchange” [All Fields]) OR “sister chromatid exchange” [All Fields]).

For chromosomal aberration: AND (“chromosome aberrations” [MeSH Terms] OR (“chromosome” [All Fields] AND “aberrations” [All Fields]) OR “chromosome aberrations” [All Fields] OR (“chromosomal” [All Fields] AND “aberration” [All Fields]) OR “chromosomal aberration” [All Fields]).

For micronucleus: AND “micronucleus” [All Fields].

For comet assay: AND (“comet assay” [MeSH Terms] OR (“comet” [All Fields] AND “assay” [All Fields]) OR “comet assay” [All Fields]).

Web of Science was searched with the mark ALL FIELDS with the words of Mexico and pesticide and then in combination, there was either comet assay, micronucleus, sister chromatid exchange, chromosomal aberration, nuclear anomalies, miRNA, or DNA damage.

Besides PubMed/Web of Science, a search was carried out for studies in Mexico on populations exposed to pesticides from 1980 to 2020 in the Redalyc and Scielo databases. Keywords such as “pesticides” and “Mexico” were used using humans as a filter, finding in total either 697 (PubMed/Web of Science), 398 (Redalyc), or 115 (Scielo) references, respectively.

**Figure 1:**
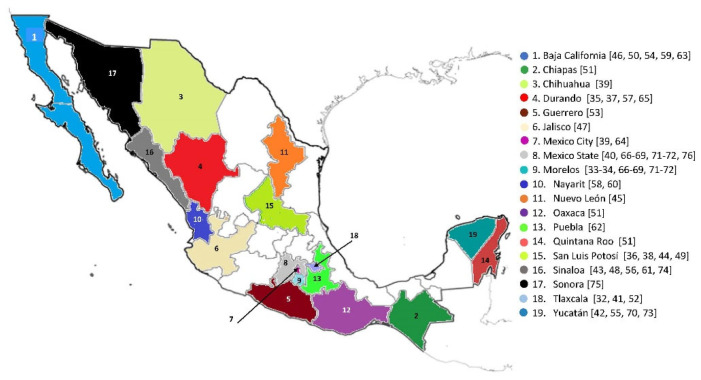
The map of the territories in Mexico where populations exposed to pesticides were investigated with genotoxic and related biomarkers.

The results were reviewed and only those that evaluated genotoxicity and related biomarkers were selected, duplicate references were eliminated and only 63 were selected, which were complemented by 5 works cited elsewhere for a total of 68 studies considered in this review in relation to the analysis of genotoxicity and related biomarkers in populations exposed to pesticides in Mexico. The map of the territories in Mexico where exposed populations were investigated is shown in Figure 1. Table 1 shows the list of the manuscripts dealing with genotoxic biomarkers, while Table 2 represents the manuscripts dealing with biomarkers of effect and susceptibility.

## 3. Results

Development, validation and the use of biomarkers as information tools for the evaluation of risk factors associated with exposure to environmental agents (such as pesticides) increases every day. There is a need to improve our knowledge concerning the adverse effects due to occupational and/or environmental exposure and lifestyle. The term biomarker includes any measure that reflects an interaction between a biological system and a potentially dangerous environmental agent, which can be physical, chemical, or biological. They can be used to identify the causes and make a better quantitative estimate of those associations at relevant exposure levels. The measurement of the response can be physiological, biochemical, and cellular or molecular level. These may also allow the identification of susceptible groups or individuals with higher or lower risk due to exposure to certain types of environmental and/or occupational agents [77].

Analysis of tissues and body fluids for the determination of compounds, their metabolites, enzymes, and other biochemical factors have been used to document the interaction of chemical agents with biological systems. The measurement of these substances, now referred as “biomarkers” are recognized as information providers that relate to exposure, internal dose, and effects, being relevant in the risk assessment process.

Biomarkers have an impact on the study of environmental risk factors. The basic interest of scientists is to explore these aspects with the purpose of predicting or preventing diseases. In risk assessment, biomarkers can be used in the identification of hazards, the assessment of exposure and to associate a response with the probability of the emergence of a disease. In general, biomarkers have been classified into three categories: exposure, damage or effect and susceptibility; however, it has been recognized that it is often impossible to clearly separate each of them, being able to overlap on some occasions [78].

The biomarkers of effect are usually used together with other biomarkers to improve our understanding of an adverse outcome, since an ideal biomarker of effect has a known mechanism that links the marker and an adverse outcome (as an adverse outcome pathway/mode of action of the chemical and the causal/correlative relationship of biological events between the marker and the adverse outcome). According to EPA, they can be divided into: (a) bio-indicators that provide a high degree of confidence in predicting the potential for adverse effects in an individual or population (such as the toxicity due to acetylcholinesterase inhibition in red blood cells); (b) markers providing a more limited and uncertain indication of the potential for adverse effects, because the events or deterministic linkages in an adverse outcome pathway are less well known (such as oxidative stress); and (c) suboptimal effects biomarkers that do not directly capture the contribution of additional factors (intrinsic and extrinsic) that may influence the incidence or severity of an adverse outcome (exogenous surrogate) [79].

### 3.1. Biomarkers of Exposure

The biomarkers of exposure include the measurement of the internal dose in biological matrices. The recent exposure of an individual is measured by the biological monitoring of human tissues and body fluids. It can be an exogenous compound (or a metabolite) within an organism, or the product of an interaction between it and some molecule or target cell, which reflects exposure to a xenobiotic. Potential exposure to pesticides from the environment can be estimated by environmental monitoring. Pesticides and their metabolites can be evaluated in biological samples of serum, fat, urine, blood, breast milk, and even exhaled air by usual analytical techniques [80].

The exposure biomarkers are divided into two subgroups: selective and non-selective, based on the specificity of the screening tests. Selective biomarkers are based on the direct measurement of the toxic or its metabolites in biological fluids (for example, blood lead levels) and non-selective biomarkers constitute a group of non-specific indicators of exposure, for example, thioethers in urine as exposure indicators to electrophilic substances and, therefore, reflection of the absorption of mutagenic and carcinogenic substances [80].

Biomarkers of exposure allow to measure biochemical, physiological, behavioral, or other alterations within an organism, depending on exposure to a xenobiotic, and that can be associated with a risk to health [78]. Biological events detected may represent variations in the number, structure, or function of cellular or biochemical components [77].

Different biomarkers are used to detect the consequences of exposure to pesticides before adverse clinical effects occur. As an example, we can mention modifications in the blood cells composition, alterations in enzymatic activities, appearance of DNA adducts, localized mRNA increase, increase of certain proteins, and even the appearance of specific antibodies against a xenobiotic or against cellular fractions (nucleus, membrane, etc.) [80], as well as other DNA damage biomarkers (genotoxicity), to which we will refer later.

In Mexico, as biomarkers of exposure, pesticide residues, especially organochlorine (OCP) and among the most important in the past dichlorodiphenyldichloroethane or DDT, have been found in the human abdominal and breast adipose tissue, human semen, blood serum, umbilical blood, serum, and breast milk, among inhabitants living in protected tropical areas [81,82,83,84,85,86,87,88,89,90,91,92]. These pesticides were also important, as they protected human and life stock from malaria. Pesticides were also used to control ectoparasites in cattle and domestic animals, as well as to eliminate scorpions [93]. Calderón-Garcidueñas et al. (2018) demonstrated that due to the lipophylic character of some pesticides, residues of hexachlorobenzene (HCB); lindane; β-hexachorocyclohexane; p,p’-dichlorodiphenyl- dichloroethylene (pp’DDE); p,p’-dichlorodiphenyldichloroethane (p,p’-DDT); and o,p’-dichlorodiphenyldichloroethane (o,p’-DDT) were detected in 1485 samples of adipose tissues of dead people who lived in Veracruz City, Mexico [92]. Comparing pesticide time trend levels in adipose tissue from 1988 to 2014, they demonstrated a decrease of pesticide concentrations through the years (p,p’-DDE decreasing time trend in the first period was 1.198 mg/kg on lipid base per year, and for the second one the decrease was 0.128 mg/kg on lipid base per year, while p,p’-DDT decreased 0.507 mg/kg on lipid base during the first period and 0.039 mg/kg on lipid base for the second). Other studies in Veracruz demonstrated that the huge and constant regular use of OCPs resulted in their high accumulation and persistence in total environment, foods, and human tissues [81,82,86,94]. People and aquatic organisms were exposed to the pesticides also from air exposure [90,95]. In Yucatan, Mexico, the pesticide concentration in the blood of women [endosulfan I (7.35 µg/mL), aldrin (3.69 µg/mL), 4,4′ DDD (2.33 µg/mL), 1.39 and 1.46 µg/mL of delta (δ)-HCH, dieldrin (1.19 µg/mL), and 1.26 µg/mL of 4,4′ DDE; In the metropolitan area, 0.080 µg/mL of gamma (ɣ)-HCH and 0.064 µg/mL of heptachlore were detected] was connected to uterine cervix cancer [22]. OCP residues were found in 24 samples of breast milk of Mayan women [30] (18.44 mg/kg of heptachlor epoxide and 1.92 mg/kg of endrin in the metropolitan zone; 2.10 mg/kg of dieldrin, 0.117 mg/kg of endosulfan II, 0.103 mg/kg of heptachlor, 0.178 mg/kg of endrin, and 0.127 mg/kg of endrin aldehyde in the main agricultural zone and on the west coast) [30]. The source of contamination was the use of water directly from contaminated wells and sinkholes [30]. In both studies, the authors detected and measured endosulfan I, II and endosulfan sulphate, but the most representative results were mentioned here.

Positive results in breast milk were also found in the Chelem (northern coastal village of Yucatan) (high concentrations of DDT, chlordanes, and lindanes, in decreasing order) [28], Kanasin (municipality joined together Merida city in a conurbation) (concentrations of lindane, chlordanes, drines, endosulfan, and DDT at approximately 1 ng/mL, and extremely high concentrations of heptachlor epoxide of 18.43 mg/kg) and Merida (heptachlor and lindanes were detected at 64–80 ng/mL) [30].

Similar results were found in the water of the Ring of Cenotes, in the area of the Yucatan aquifer. Heptachlor, endrin, endrin aldehyde, endosulfan sulfate, DDT and its metabolites were detected in the aquifer of Yucatan at concentrations ranging from 457 to 10 864 ng/mL [7]. In the air samples of the industrial zone of Merida, the capital of Yucatan, pesticides found were mainly degradation products of DDT such as DDD and DDE and also DDT itself, endosulfan, heptachlor, and isomeric forms of heptachlor epoxide. The pesticide contamination sources were from karstic soil that had high vulnerability to groundwater pollution due to the use of OCPs in agriculture and livestock activities, and with the population’s poor knowledge about the safe use of pesticides (Mayan) [93]. Contamination was also from home use pesticides for family gardens, agriculture and campaigns to control vector-borne diseases (DDT), such as in termite control (chlordane), or ectoparasites on cattle and domestic animals or to eliminate scorpions (lindane) [93]. The use of heptachlor for termite control was prohibited in Mexico in 2003, but with approximately 213 tons/year already contaminating the land from 1992–1993 [96], and with lindane use still allowed according to the North American Commission for Environmental Cooperation [97], and Mexico importing until 2016, 20 tons/year of lindane for lice extermination and ectoparasite control in domestic animals. Fourteen years ago, Mexico was in the top ten countries with the highest technical and public health OCPs uses [98].

The levels of blood cholinesterase can also be used for pesticide exposure assessment, including organophosphates and carbamates exposure. These enzymes [acetylcholinesterase (AChE) and butrylcholinesterase (BuChE)] are required in helping the human body function by breaking down the neurotransmitter acetylcholine [99]. There are also fingerstick ChE tests available as a quick, minimally invasive and portable way of sampling and testing.

Studies in agricultural workers from several regions of the country reported moderate to severe cholinergic symptoms, including decreased AChE [100]. Using this type of testing, Trueblood et al. (2019) examined 54 adolescents living along the Texas-Mexico border, where adolescents often engage in farm work [101]. Results demonstrated 3.75 U/mL (95% CI 3.51–3.98) mean AChE value in males, while females had a mean AChE value of 2.86 U/mL (95% CI 2.64–3.08), which was statistically significant. However, Payán-Rentería et al. (2012), and Alvarado Ibarra et al. (2019) did not demonstrate different AChE values among agricultural workers when compared to the control group [47,75]. Similarly, no difference was found for the BuChE levels between exposed and controls in southeastern Mexico by Ruiz-Gamboa et al., 2018 [73].

Besides the examination of acetylcholinesterase levels, levels of hemoglobin, transaminases and alkaline phosphatases were examined by Galindo-Reyes and Alegría (2018), in a cohort study on 8 farms in the Navolato Valley, Sinaloa, Mexico. This municipality has the largest number of migrant farmworkers, (over 20% of the population) mainly from the states of Oaxaca, Guerrero, Zacatecas and Durango [74]. Migration was mainly due to the large demand of seasonal farmworkers for the cultivation and harvesting of vegetables and grains. They found 17 OCP and 9 organophosphorous pesticides (OP) in soil and water samples [74]. Among them, for 14 OCPs and 6 OPs similar concentrations were also found in the blood and urine of 49 farmworkers [74]. Those farmworkers had elevated levels of transaminases and alkaline phosphatase, while levels of hemoglobin and acetylcholinesterase were decreased when compared to the control group (20 people). The results indicated that the farmworkers suffered from chronic exposure to workplace pesticides and could endure adverse health effects [74].

Martínez-Valenzuela et al. (2017a) demonstrated that serum lipid OCPs concentrations represent an important indicator for both human biological matrices [102]. The authors measured higher pesticide concentrations in 126 pair samples of adipose tissue and blood serum during autopsies from Los Mochis Sinaloa, Mexico (higher concentrations correspond to b-HCH, pp’DDE and op’DDT in blood serum lipids; and pp’DDT shows higher concentrations in adipose tissue), with a significant linear association of lipid serum organochlorine pesticide concentrations with that in adipose tissue [102].

From National Health and Nutrition Examination Survey (NHANES) 1999–2004 (unweighted *n* = 1411, population estimate = 3,760,609) data, Everet et al. (2017), examined the blood of Mexican American teens, 12–19 years old (19.8% of the data), born in Mexico, who were exposed to DDT before they immigrated to the US (DDT was banned in Mexico in 2000) [103]. They found that levels of p,p’-DDT > 0.086 ng/g were associated with total diabetes with nephropathy (odds ratio = 4.42, 95% CI 2.23–8.76), and with total diabetes without nephropathy (odds ratio = 2.02, 95% CI 1.19–3.44). The third quartile of p,p’-DDE (2.99–7.67 ng/g) and the fourth quartile of p,p’-DDE (>7.68 ng/g) were associated with diabetic nephropathy and had odds ratios of 5.32 (95% CI 1.05–26.87) and 14.95 (95% CI 2.96–75.48) compared to less than the median, respectively [103]. p,p’-DDE was not associated with total diabetes without nephropathy [103].

The biomonitoring of pollutants in contaminated areas is a high-priority health issue for children residing in such places. In their study, Flores-Ramírez et al. (2017) determined the levels of different organic pollutants (HCB, α-endosulfan, β-endosulfan, endosulfan sulfate, DDE and PCB 101) in children aged 6 to 12 years from high-risk both indigenous and industrial areas [104]. They found that the children living in contaminated sites presented the highest levels of persistent organic pollutants (POPs) in serum [104]. Contributing to the knowledge of exposure to POPs in Mexico, authors demonstrated that the handling of toxic substances continues to be inadequate, representing a risk for humans and the ecosystem in general [104]. Exposure to complex mixtures of pollutants is a public health problem, and children are one of the populations most susceptible to these effects. Therefore, more studies are needed to develop and implement intervention programs that reduce health risks in communities at risk of exposure. Children not working in agriculture and living in Ciudad Juarez, Chihuahua, Mexico (a major manufacturing center in Mexico) demonstrated higher levels of different pesticide biomarkers in urine and blood, probably due to the consumption of farm products [105].

Serum levels of thyroid hormones in floriculture workers were also analyzed in connection with exposure to p,p’-DDE (a stable metabolite of DDT) in the longitudinal study on 136 male subjects from the State of Mexico and Morelos, Mexico [72]. Workers were occupationally exposed to pesticides, during agricultural periods of high (rainy season) and low (dry season) levels of pesticide application [72]. There were geometric means of p,p’-DDE levels of 6.17 ng/mL and 4.71 ng/mL in the rainy and dry seasons, respectively [72]. Blanco-Muñoz et al. (2016) also observed positive associations between the serum levels of p,p’-DDE and those of total triiodothyronine (T3) (β = 0.01, 95% CI: −0.009, 0.03), and total thyroxine (T4) levels (β = 0.08, 95% CI: 0.03, 0.14) and negative, but no significant changes, in thyrotropin (TSH) levels in male floricultural workers, supporting the hypothesis that pesticides can act as a thyroid disruptor in humans.

Watkins et al. (2016) examined the association between maternal urinary 3-phenoxybenzoic acid (3-PBA) concentrations during the third trimester of pregnancy as a measure of in utero pyrethroid exposure to the fetus [106]. Participants were part of an established Mexico City birth cohort (187 participants), with children’s scores on the Mental Development Index (MDI) and Psychomotor Development Index (PDI) from the Bayley Scales for Infant Development (BSID-IIS) at 24 and 36 months of age of children [106]. In their study, 3-PBA was detected in 46% of all urine samples, with similar detection rates and geometric mean concentrations across pregnancy among the 21 participants who provided repeat samples. Participants in the medium and high 3-PBA categories (>LOD) had lower MDI scores at 24 months compared to those in the low 3-PBA category (<LOD) after adjustment for covariates (*p*-value trend = 0.07), with slightly stronger associations among female children. The three-level categorical variable for the third trimester in utero 3-PBA was not associated with MDI scores at 36 months or with PDI scores at either time point.

Herrero-Mercado et al. (2011), determined the levels and calculated ratios of copartition coefficients among OCPs beta (β)-HCH, pp’DDE, op’DDT and pp’DDT in maternal adipose tissue, maternal blood serum and umbilical blood serum on a fat basis among 70 mother-infant pairs from Veracruz, Mexico [89]. p,p’-DDE was the major OCP component, detected in every maternal adipose tissue (0.770 mg/kg), maternal serum sample (5.8 mg/kg on fat basis) and an umbilical cord blood sample (6.9 mg/kg on fat basis). p,p’-DDT was detected at 0.101 mg/kg, 2.2 mg/kg and 5.9 mg/kg, respectively, according to the order given above. β-HCH was detected at 0.027 mg/kg, 4.2 mg/kg and 28.0 mg/kg respectively. op’DDT was detected only in maternal adipose tissue at 0.011 mg/kg. The copartition coefficients among samples identified significant increases in concentrations from adipose tissue to maternal blood serum and to umbilical blood serum. The increase indicated that maternal adipose tissue released OCPs to blood serum and that they were carried over to umbilical cord blood.

O’Rourke et al. (2000), examined urine samples of 154 children of 6 years of age living in a heavily farmed border (US-Mexico) community, since they live closer to the ground and take in greater amounts of food relative to body mass than older children or adults [107]. They detected diethylphosphates (DEPs) and dimethylphosphates (DMPs) above the reference range for 1000 non-occupationally exposed individuals (DL = 25 µg/g creatinine, Cr), with at least one metabolite detected for 33% of the subjects and many samples contained multiple biomarkers. DEP was detected in 5% of the subjects, with no seasonal creatinine differences for six-year-olds. The urinary OP screen was effective in identifying subjects with atypical internal doses.

Pesticide aerial application is the most frequent source of exposure [108], causing exposure of agricultural workers involved directly in agricultural practices and inhabitants living in neighboring communities near pesticide-sprayed fields [109]. Their direct application on fields is usually to protect plants from plagues, thereby increasing agricultural productivity and crop yields [1]. Airplanes are often used for pesticide spraying on fields, but also in malaria control. Martínez-Valenzuela et al. (2017) only examined DNA damage among 30 pilots (results explained in biomarkers of DNA damage) but did not examine the levels of pesticides in the air [56]. Throughout Mexico, the OCP levels in the air were determined by Wong et al. (2009), and in all monitored sites, the most abundant were DDT and its metabolites [110]. Northern and central Mexico had lower concentrations compared to southern Mexico, an endemic malarious area of the country, showing ΣDDT ranging from 15 to 2360 pg/m^3^ [110]. On the other side, due to the local usage, higher concentrations of ΣENDO (26,800 pg/m^3^) were registered at an agricultural area in Mazatlan, Sinaloa, during the winter and spring periods [110]. A differential observed distribution of the OCPs also depended on their aged or diffusion capacity, as well as their intensive use in agricultural or malarious regions [110].

Reports from the news media have suggested that intensive pesticide usage in agriculture has resulted not only in acute intoxication cases but also in chronic diseases among farmworkers and populations near farmlands [111].

Since OCPs are continuously circulating and equilibrating among body compartments due to their hydrophobic nature, persistence, and accumulation in tissues rich in lipids, Caba et al. (2015), examined their levels in blood serum and in total lipid contents in Veracruz, México inhabitants [112]. From one tertile to the next β-HCH, authors showed a decrease of −3.19 mg/kg on lipid basis, pp’DDE level decrease by −3.70 mg/kg on lipid basis and pp’DDT level decrease −1.13 mg/kg on lipid basis [112]. They concluded that the levels and the orderly sequence of OCPs distributions in the blood serum maintain an inverse relationship to total lipid blood serum concentrations [112].

In the Lower Rio Grande Valley of Texas, Sexton and Salinas (2014) investigated concentrations of OCPs/metabolites, polychlorinated biphenyls (PCBs), and polycyclic aromatic hydrocarbons (PAHs) measured in maternal and umbilical cord blood from pregnant Hispanic women in Brownsville, TX [113]. Results demonstrated that both mothers and fetuses were exposed concurrently to a variety of relatively low-level, hazardous environmental chemicals. Approximately 10% of the blood specimens had comparatively high concentrations of specific OCPs, PCBs and PAHs. Because many pregnant women in Brownsville live in socio-economically-disadvantaged and environmentally challenging circumstances, there is appropriate concern that exposure to these exogenous substances, either individually or in combination, may contribute to endemic health problems in this population, including cardiovascular disease, obesity, and diabetes. The challenge is to identify individuals at the highest comparative risk and then implement effective programs to either prevent or reduce cumulative exposures that pose significant health-related threats.

### 3.2. Susceptibility Biomarkers

In this type of study, it is important to consider different variables that may be affecting the response, so it is necessary to determine, in addition to the classical biomarkers of exposure and/or damage, so-called susceptibility biomarkers. They are constituted mainly by genetic polymorphisms that refer to the individual differences in their metabolic capacity, making individuals more or less susceptible to damage by exposure to certain agents such as pesticides.

Although individuals may experience similar environmental exposures, genetic characteristics may produce marked differences in the target site and thus a different level of response. Susceptibility biomarkers may reflect genetic or acquired factors that influence responses to exposure [78]. These factors are pre-existing and independent of exposure; they are predominantly of genetic origin, although diseases, physiological changes, medication, and exposure to other environmental factors, can also alter individual susceptibility.

The biomarkers of susceptibility serve as indicators of the individual response to the aggression of a xenobiotic. The importance of these biomarkers lies in their consideration of interindividual variations (differences in absorption, bioavailability, excretion, or DNA repair mechanisms). Two types of susceptibility biomarkers can be distinguished: activating systems and detoxifying systems. In the case of the former, we can mention the cytochrome P450 system, implicated in the toxicity of numerous xenobiotics. Enzymes such as glutathione S-transferase (GST), N-acetyltransferase 2 (NAT2), sulfotransferase, glucuronyltransferase, or paraoxonase 1 (PON1) are involved in the second group [80].

#### 3.2.1. Effect of Genotypes on Cytogenetic Damage

The genotypes responsible for interindividual differences in the ability to activate or inactivate genotoxic agents are recognized as biomarkers of susceptibility. It has been suggested that different enzyme isoforms contribute to individual susceptibility as genetic risk modifiers for cancer after exposure to genotoxic agents. Within these modifiers, the following genes have been implicated in the metabolism of pesticides: cytochrome P450 E21 (CYP2E1); GST; NAT2; and PON1 [1].

Paraoxonase 1 (PON1) is an enzyme-linked to high-density proteins that decreases lipid peroxidation and is also involved in the detoxification (deactivation) of some OPs [114].

Pérez-Herrera et al. (2008) associated the genetic polymorphism PON1Q192R with poor semen quality [according to WHO standards and DNA integrity (sperm DNA damage evaluated by in situ-nick translation (NT-positive cells)] [42]. In a cross-sectional study on 54 agricultural workers exposed to OP (18–55 years old) from the state of Yucatan (Mayan ascendancy, southeast Mexico) in the month of sampling and during three months before sampling (spermatogenic cycle spermatids-spermatozoa), PON1 192R and 192Q allele frequencies were 0.54 and 0.46, respectively [42]. In relation to DNA integrity, homozygous subjects for the 192R allele presented a dose-effect relationship with exposure to OO during three months before sampling, being more susceptible than the subjects carrying the Q allele. Furthermore, within a month of sampling, OP exposure and NT positive cells and sperm viability were connected with the same genotype. They also suggest that the cells in the latter stages of maturation and probably in the first of spermatogenesis are more sensitive to OP toxicity, but also that cells at all stages of spermatogenesis are OP targets, and that there is an interaction between OP exposure and PON1Q192R polymorphism on these effects; farmers featuring the 192RR genotype were more susceptible to develop toxic reproductive effects by OP exposure.

In order to evaluate the influence of PON1 polymorphisms on the activity of this enzyme, a cross-sectional study was carried out with 170 floriculturists from the states of Morelos and Mexico [67]. With a minimum of 6 months of exposure to pesticides, 50 of them practiced organic floriculture [67]. The PON155 polymorphism has no significant effect on the serum activity of PON1 in any substrate. However, they found a significant variation in PON1 activity on paraoxon and diazoxon depending on the polymorphisms PON1 192 and PON1-108. The genotypes 192RR and −108CC demonstrated a greater and the genotypes 192OO and −108TT lower paraoxonase activity. However, the 192RR genotype has the lowest diazoxonase activity, while the 192QQ genotype has the highest activity against this substrate.

Torres-Sánchez et al. (2019) studied 381 healthy pregnant women (< 17 gestational weeks) living in a floricultural region of Mexico where pesticides, including OPs, are routinely used and genotyped for the paraoxonase gene SNP polymorphisms (PON1192QR, PON155LM and PON1-108CT) [76]. Their results demonstrated that 162 women (42.52%) were para-occupationally exposed to pesticides. Although there were no significant interactions observed between the pesticides’ para-occupational exposure and PON1 polymorphisms, independently of para-occupational exposure, the likelihood of hypothyroxinemia was higher among women who were carriers of PON155MM than in those with the PON155LL genotype (OR MM vs. LL: 3.03; 95%CI 1.62, 5.70). PON1192 RR (OR RR vs. QQ: 1.72; 95%CI 0.93, 3.17) and PON1-108TT (OR TT vs. CC: 1.60; 95%CI 0.90, 2.70) genotypes were marginally associated with hypothyroxinemia. From the same area, male flower workers demonstrated a positive association between urine concentration of dialkylphosphate (DAP) OP metabolites and TSH and total T4 levels and a negative association with total T3 [68]. Lacasaña et al. (2010) made a longitudinal study on a population of floriculture workers from Mexico, during two periods of high and low-intensity levels of pesticide application [69]. They found a significant interaction between serum diazoxonase activity and total dialkylphosphates (ΣDAP) on TSH levels. Thus, when PON1 activity was increased, they observed a decrease in the percentage of variation of TSH levels for each increment in one logarithmic unit of ΣDAP levels. This interaction was also observed with the PON1192RR genotype. These results suggest a stronger association between OP and thyroid function in individuals with lower PON1 activity. Interestingly, among the individuals with the same genotype, PON1 activity was able to vary up to 13 times [115].

Placental oxidative stress has been involved in the pathogenesis of certain reproductive adverse effects, including miscarriage. Paraxonase 1 (PON1) is a high-density lipoprotein (HDL)-linked enzyme that prevents the oxidation of low-density lipoproteins (LDL) and as A-esterase PON1 is capable of hydrolyzing the active metabolites (oxons) of a number of OP insecticides such as parathion, diazinon and chlorpyrifos. Evidence is slowly emerging that a low PON1 status may increase susceptibility to OP toxicity in humans [116].

Fortenberry et al. (2014) conducted a study including 264 women (floriculturists and wives of floriculturists) who had been pregnant sometime during the 10 years preceding the study to examine whether maternal and/or child PON1 genotypes (PON1R192Q and PON1L55M) were associated with Attention Deficit Hyperactivity Disorder-Like Phenotypes (ADHD-LP) in a Mexico City, Mexico birth cohort [117]. Significant associations were observed with maternal genotypes but not with child genotypes. A higher DSM IV Hyperactivity/Impulsivity score [β = 3.27 points; 95% CI (0.89, 5.65)] and a 2.17 higher score in child DSM IV Total [95% CI (0.05, 4.29)] were observed for maternal PON155MM in comparison with PON155LMþLL. The child attention problems score was 2.27 points higher [95% CI (0.002, 4.53)] for maternal PON1192QQ in comparison to PON1192QRþRR. Because maternal PON1 polymorphisms were associated with child ADHD-LP, this may be a viable biomarker of susceptibility for ADHD-LP.

Moreno-Banda et al. (2009) determined the association between maternal exposure to floriculture during pregnancy and the risk of low birth weight (LBW) (2500 g) in their offspring, together with the interaction between this exposure and maternal genotype for PON1 Q192R polymorphisms [66]. A cross-sectional study was carried out in two Mexican states (State of Mexico and Morelos) with high frequencies of greenhouse activity. Blood samples were collected from 264 females (floriculturists or partners of floricultural workers) who became pregnant during the 10 years prior to the interview. Information was obtained pertaining to 467 pregnancies. After adjusting for potential confounders, they detected a statistically significant interaction between maternal exposure to flower growing work during pregnancy and PON1 Q192R polymorphisms on risk of LBW. The risk of having a baby with LBW is nearly six times higher if a mother is a floriculture worker during pregnancy and has the PON1 192RR genotype (OR 5.93, 95% CI 1.28, 27.5). These results suggested that the interaction between maternal floriculture work during pregnancy and the 192RR PON1 genotype increased the probability of having children with LBW.

Blanco-Muñoz et al. (2013), in their cross-sectional study on 514 pregnancies (35 miscarriages and 479 controls), demonstrated that the risk of miscarriage by mothers with PON1192RR genotype was 2.2 higher than by mothers with the PON1192QR/PON1192QQ genotype (95% CI 0.93–5.17) [71]. The risk was close to 4 times higher in mothers with PON155MM/PON155LM genotype than in mothers with PON155LL genotype (OR = 3.9; 95% CI 1.38–11.0). No significant differences were found in risk of miscarriage based on the maternal PON1-108C/T genotype. No evidence was found of an interaction between the various PON1 genotypes and the mothers’ floricultural activity during pregnancy. This study suggests an effect of genetic maternal PON1 polymorphisms on miscarriage and provides additional evidence that combines with the growing information about the ways in which certain PON1 genotypes can affect the development of the fetus in utero.

González-Herrera et al. (2010), made a case-control study on 160 control parents and 152 children with spina bifida, a common congenital malformation in Southeast Mexico in children from parents in areas with frequent pesticide spraying or agriculture activities, suggesting potential exposure to pesticides [70]. The frequency of PON1 haplotypes and polymorphisms (2108CT, L55M, and Q192R) were determined in the study. The genotype frequencies for the three PON1 polymorphisms distributed according to Hardy–Weinberg expectations (*p* > 0.05) were significantly different between cases and controls (*p* < 0.05). The heterozygous CT genotype of 2108CT polymorphism, the RR genotype of Q192R polymorphism, both LM and MM genotypes of L55M polymorphism, and the haplotypes 221 and 222 (for 2108CT, L55M, and Q192R) were associated with the risk of having a child affected by SB (*p* < 0.02). The heterozygous 2108CT genotype was associated only maternally, whereas the heterozygous L55M genotype was relevant only in the fathers. The RR homozygous genotype was relevant both in mothers and fathers, suggesting the importance of this substrate-specific polymorphism. Results suggested that PON1 polymorphisms are relevant risk factors in this population from Southeast Mexico for having offspring affected with SB.

López-Flores et al. (2009) reported in their cross-sectional study on the distribution of three common genetic polymorphisms of the PON1 gene in a population of floriculture workers from Mexico that genotype frequencies at position PON155 were 89% (LL), 10% (LM) and 0.6% (MM), at position PON1192 16% (QQ), 47% (QR) and 37% (RR), and 26% (TT), 42% (TC) and 32% (CC) at position PON1-108 [67]. Thus, the frequencies of alleles L, Q and T were 0.94, 0.40 and 0.47, respectively. The PON155 polymorphism had no significant effect on serum PON1 activity on any substrate. They found a significant association between the PON1192 polymorphism and PON1 activity towards paraoxon and diazoxon, which increased in genotypes as follows: 192RR > 192QR > 192QQ for paraoxonase activity and, inversely, 192QQ > 192QR > 192RR for diazoxonase activity. The PON1-108 polymorphism also had a significant effect on PON1 activity level towards paraoxon in the following order among the genotype groups: −108CC > −108TC > −108TT. Serum PON1 activity towards diazoxon was not associated with the PON1-108 polymorphism, but it was influenced by the intensity exposure to pesticides at the floriculture industry and years of occupational exposure to pesticides. No polymorphism significantly influenced serum PON1 activity on phenylacetate.

Rojas-García et al. (2005) conducted a study in order to evaluate PON1 phenotype and the frequencies of polymorphisms PON1 162, 108, 55, and 192 in a Mexican population in 214 unrelated individuals of both gender, 18–52 years old [64]. They found a wide interindividual variability of PON1 activity with a unimodal distribution; the range of enzymatic activity toward phenylacetate was 84.72 to 422.0 U/mL, and 88.37 to 1645.6 U/L toward paraoxon. All four PON1 polymorphisms showed strong linkage disequilibrium (D% N90). PON1 polymorphisms 108, 55, and 192 were independently associated with arylesterase activity; whereas the activity toward paraoxon was related only with the PON1 192 polymorphism, suggesting that this polymorphism is key to inferring PON1 activity.

Montero et al. (2006) studied industrial workers, agricultural workers and workers in households, education, and commerce as three occupations exposed to chemical exposure in a transition zone from rural activities towards intensive industrialization in the environmentally polluted zone of the Atoyac and Xochiac rivers [41]. The individuals were also genotyped for polymorphisms of glutathione transferase mu1 (GSTM1) and theta1 (GSTT1), which are relevant when oxidative responses are involved. The genotoxic damage evaluated by micronucleus assay was differentially distributed in the regions studied, being more affected in those closer to the Atoyac and Xochiac rivers, indicating an effect due to environmental exposure to the contaminants present in the rivers. Increased genotoxic damage was found, including cells with >1 MN, >1 chromatin bud, and nucleoplasmic bridges.

#### 3.2.2. Circulating DNA Quantification

The precise clinical and physiopathological significance of detecting plasma circulating DNA fragments is still under investigation. Some authors have hypothesized that DNA fragments in cancer patients can induce metastasis development, probably by acting on susceptible normal cells [118]. Among different physiological biomarkers, Payán-Rentería et al. (2012) found a statistically significant difference of this parameter between farmworkers and the control group belonging to the Nextipac community in Jalisco, Mexico [47].

#### 3.2.3. miRNAs-Possible New Biomarker of Susceptibility

Different epigenetic mechanisms, including the expression of microRNAs (miRNA), can change the function of the genome under exogenous influence. Thus, pesticides can alter gene regulation and act on human health. The miRNAs play important roles in the regulation of a wide variety of cellular and biological processes including growth, development, differentiation, proliferation, cycle control and cell death.

Recently, new approaches aimed at evaluating the mechanisms by which pesticides can alter gene regulation and act on human health have been developed. Among these new approaches, epigenetics seems to be a promising tool. However, until today the specific molecular mechanisms that relate exposure to health effects have not yet been established.

The effects of pesticides on the epigenome can be attributed to changes in the expression profiles of the miRNAs. The identification of the specific binomials miRNA-chemical agent (pesticide) will not only help the understanding of environmental diseases but can open the way for new strategies of biomonitoring and prevention.

In this context, Valencia-Quintana et al. (2018) proposed to validate the alterations in the expression profiles of the miRNAs as a potential biomarker of exposure to pesticides. The establishment of its relationship with possible damage to health in people exposed to these compounds will be of great importance in the near future [119].

With the functions of miRNAs identified in almost all aspects of biological processes, their role as major mediators of cellular response to extracellular signals, as well as regulators for the proper control of tissue homeostasis may be more relevant than was suspected previously. The growing evidence that the expression of miRNAs is affected by known toxic substances, as well as by oxidative stress and other forms of cellular stress, certainly suggests an important role of these in toxicology, which could provide a link between the influences of the environment and gene expression.

The effects of pesticides on the epigenome can also be attributed to changes in the expression profiles of miRNAs, producing changes in gene regulation which may explain the harmful effects that these compounds have on human health.

However, until today there have been few studies that address the binomial miRNAs-pesticides and there are no reports of these in occupational or environmentally exposed populations.

Identifying the specific binomials miRNA-chemical agent, in this case, pesticides, will not only help the understanding of environmental diseases, but can pave the way for new biomonitoring and prevention strategies. Thus, it is critically important to be able to identify and validate miRNAs that can be induced by specific environmental compounds (pesticides). Future studies will be necessary to demonstrate the contribution of environment-miRNA interactions to human diseases.

### 3.3. Biomarkers of Genotoxicity

Genotoxicological monitoring in human populations is a very useful tool for the estimation of genetic risks derived from environmental exposure to complex mixtures of potentially genotoxic agents [3,4]. Genotoxic potential is a primary risk factor for long-term effects such as carcinogenesis and reproductive toxicology. Most pesticides have been evaluated in a wide variety of mutagenicity tests such as gene mutations, chromosomal aberrations (CA) and DNA damage. Although it is difficult to establish a connection between exposure to pesticides and the prevalence of cancer, especially due to the large number of compounds involved, some authors demonstrated a higher incidence of certain types of cancer in populations exposed to pesticides [120]. On the other hand, studies of genotoxic damage of this type of compound have been conflicting since some indicate significant increases in the frequencies of damage, while others do not present significant differences [3,4].

The early detection of genetic damage allows taking the necessary measures to reduce or suppress exposure to the deleterious agent when it is still reversible, and thus prevent and reduce the risk of developing pathological changes, such as cancer.

The determination of chromosomal alterations is an established method for monitoring populations occupationally or environmentally exposed to potentially genotoxic agents, such as pesticides. Visible damage to human chromosomes such as: chromosomal aberratios (CA), sister chromatid exchanges (SCE), micronucleus (MN), and nuclear abnormalities (NA), among others, can be detected.

On the other hand, genetic polymorphisms as susceptibility modifiers have received recent attention, and there is increasing interest in conducting studies to explore gene-environment interactions with the purpose of detecting susceptible populations prone to develop health damage by exposure to chemical agents [121].

In human monitoring studies, variations of the basal frequencies are presented due to the presence of endogenous factors (gender, age, medical history, etc.) and exogenous factors (lifestyles, smoking and drinking habits, as well as eating habits, among others), so it is important to consider the possible influence of these factors on the populations studied.

#### 3.3.1. Chromosomal Aberrations (CA)

Genetic damage at the chromosomal level leads to an alteration in the number and/or structure of the chromosomes and such an alteration can be measured as a frequency of CA. It is thought that CA originates from errors in the repair of DNA lesions. The increase in the frequency of CA is considered a biomarker of exposure and/or the effect of early damage to DNA, allowing the detection of alterations resulting from the aneugenic or clastogenic effect of xenobiotics [122]. On the other hand, there is evidence linking CAs with the risk of developing cancer. In Mexico, there was an article in 1987 from Zapata Gayón et al., who examined clastogenic changes of chromosomes in a population of individuals occupationally exposed to different pesticides, finding statistically significant increments for simple and double breaks [31]. Steenland et al. (1997) evaluated a population exposed to ethylenebis (dithiocarbamate) fungicides (EDBC), with CA as one of the cytogenetic markers [33]. The study evaluated 49 highly exposed workers, 14 slightly exposed and 31 non-exposed and found significant increases in the frequencies of chromosomal translocations in applicators with respect to the control group, within the unadjusted data, significant differences between the high and lightly exposed when compared with the control group for translocations and reciprocal translocations, however, the translocations increased markedly with age, making age as a confounder. After age adjustment, the results indicated more total translocations among applicators than in the non-exposed subjects, while the slightly exposed ones did not present significant differences. Poisson regression analysis of other variables that alter chromosomal events (dicentrics, insertions, fragments, and rings) did not present significant differences between the exposed group and the unexposed group.

#### 3.3.2. Sister Chromatid Exchange (SCE)

A reciprocal exchange of DNA between sister chromatids of a duplicated chromosome induces SCE [122]. It is considered that SCEs are cytogenetic biomarkers more sensitive, quicker, and simpler than CAs to evaluate the genotoxic potential of a variety of mutagenic and carcinogenic agents, such as pesticides. SCEs have been adopted as a genotoxicity biomarker in agents capable of interfering in the replication of DNA, although the mechanism of action that leads to its increase and its biological significance until today have been unknown. These changes are cytologically visible through the differential staining of the chromatids. Depending on the statistical sensitivity required, a minimum of 50 cells must be analyzed, with 25 cells in each of the two replicates. This test is especially useful for monitoring populations exposed to low concentrations of chemical agents.

In a rural population from Tlaxcala, Mexico, Gómez-Arroyo et al. (1992) evaluated occupational contact with pesticides in 170 men, 94 exposed and 76 not exposed [32]. They demonstrated that SCE followed a normal distribution with no differences between the two groups (*p* = 0.4, Student’s *t*-test). SCE frequency also did not correlate with the duration of exposure of the rural workers (r = −0.06), nor tobacco intake or alcohol ingestion (multiple covariance analysis). The authors attribute the negative effects found to the fact that in some cases, people were exposed to a single pesticide in each application and to a mixture, chronically but only for short periods of time each year when working on small plots of land, and the level exposure was not enough to produce SCE.

Unlike the previous study, Steenland et al. (1997), in a population of fungicide sprayers, found significant increases in SCE, as with CA in human lymphocytes, as mentioned above [33]. The unadjusted data, as well as the adjusted ones (by age and smoking habits, via linear regression) indicated that the applicators had significantly higher SCE than unexposed. Cytogenetic damage in floriculturists exposed to pesticides from the Morelos State was evaluated by the SCE test in human lymphocytes, in addition to other geno- and cytotoxic tests [34]. The non-exposed population had an average of 4.0 + 0.1, statistically different from the value of 7.1 + 0.17 SCE/cell found in the exposed population, with no correlation between the observed damage and the exposure time, due to the fact that the group with the shortest exposure time had similar frequencies of SCE when compared with those with the longest contact time. In this case, since the products of florists are not for human consumption, workers tend to use a greater quantity and higher concentrations of pesticides to improve their production. Generally, in occupational exposure to pesticides, flower growers are in contact with mixtures, which contain different active ingredients, some of which have already been banned in different countries due to their mutagenicity and carcinogenicity.

In the community of Las Grullas, Ahome, Sinaloa, a region where most of the population is exposed to pesticides, a sample of 70 agricultural workers was selected, with an average of 7 years of exposure to OP (malathion, methyl parathion, diazinon, monocrotophos and gusatión) and carbamates (aldicarb, carbaryl, carbosulfan, and lannate), during the sowing of chili, tomato, as well as mango and were compared with a control population (70 individuals) from the city of Los Mochis. The overall SCE average for the exposed group of 6.36 + 0.22 significantly differ from the non-exposed group (3.71 + 0.11, Student’s *t*-test), and the effect was significantly correlated with the time of exposure to pesticides, without differences between smokers and non-smokers, or without any relationship with the intake/or not of alcoholic beverages [43].

#### 3.3.3. Micronuclei (MN) and Nuclear Abnormalities (NA)

Another way to measure the chromosomal damage are MNs. They represent either chromosomal acentric fragments or complete chromosomes with inactivated centromeres left behind during cell division (mitosis or meiosis), which will appear in the cytoplasm during the interface as small additional nuclei. This assay, like the CA test, allows the detection of aneugenic and clastogenic agents. In general terms, the MN registry is much simpler and faster than the CA registry. Like CAs, an increase in the frequency of MN implies a risk of developing cancer [123].

In addition to MN, other nuclear anomalies (NA) such as binucleated cells, condensed chromatin, karyolysis, pykosis, karyorrhexis and nuclear buds, together with broken eggs [124], which reflects physiological alterations in the cell, can be recorded, in human blood and buccal cells. This damage may also be related to exposure to environmental agents.

In the human MN project (HUMN Project), MN were analyzed in peripheral blood lymphocytes of exposed and unexposed individuals, mainly because it was a well-established human test system at the time the project began. In this assay, the methodology has been standardized, with defined frequencies and characterization of the effects of demographic, genetic, as well as methodological factors and it has been determined if the MN frequencies in different tissues are predictors of cancer risk [123,125]. Although there are MN assays performed on human lymphocytes and rats in order to evaluate in vitro the influence and genotoxicity of different pesticides and their concentration, there are not many studies involving MN assay and human biomonitoring using human lymphocytes. In an agricultural community pesticide exposure in mother-child pairs was evaluated, quantifying 15 organochlorine pesticides, found in maternal plasma a concentration range from 5000 to 25,500 ng/g fat, by other side in the umbilical cord plasma of newborns were detected in a range from 9800 to 285,500 ng/g fat. Regarding clastogenic damage, a range of MN and nuclear anomalies from 0.33 to 5 and from 0 to 8 was obtained in mothers and newborns, respectively, while in the unexposed population from 0 to 3. Although the levels of organochlorine pesticides in newborns were significantly higher than in mothers, the differences in DNA damage did not show statistically significant differences. Most mothers take folic acid, which could prevent genetic damage, even though fetuses were exposed to pesticides during development [39,44,49], showing that the MN test in umbilical cord samples might be useful in the evaluation of transplacental genotoxic agents and can be compared with the DNA damage in their mothers after their study in Chihuahua, Mexico. Newborns and mothers from urban areas showed 1 ± 0.9 and 3.7 ± 1.4 MN in 1000 cells, respectively.

On the other hand, newborns and mothers from the agricultural area presented a frequency of 2 ± 1.5 and 4.5 ± 2.4 in 1000 cells, respectively. Montaño-Soto et al. (2014) measured MN frequency in 26 women occupationally exposed to pesticides from Maneadero Valley, an important agro-industrial region in Baja California, Mexico, using genotoxic biomonitoring and 22 controls [50]. Exposed women revealed significantly elevated frequencies of micronuclei (Mann–Whitney U-Test, *p* < 0.05) compared to the control group, with no statistically significant differences in nuclear index and chromatin bridges. The cluster analysis showed a strong relationship between micronuclei and exposure, suggesting that genotoxicity is associated with occupational exposure to agrochemicals.

Montero et al. (2006) examined a complex situation of chemical exposure in a transition zone from rural activities towards intensive industrialization, which brought environmental pollution in the Atoyac and Xochiac rivers [41]. Three occupations were distinguished, according to chemical exposure: industrial workers, agricultural workers and workers in households, education and commerce. Increased genotoxic damage was found, including cells with > 1 MN, > 1 chromatin bud, and nucleoplasmic bridges. The genotoxic damage was differentially distributed in the regions studied, affecting more those that are closer to the Atoyac and Xochiac rivers, indicating an effect due to environmental exposure to the contaminants present in the rivers. Xotlanihua-Gervacio et al. (2018) conducted a cross-sectional study with 201 individuals, some of whom were dedicated to the spraying of pesticides and stratified them into three groups: 23 individuals who were not in direct contact with pesticides, 120 individuals as a moderately exposed group and 58 people with high exposure [60]. The geometric mean (GM) of MN was 5.4 (1–26 MN). The results demonstrated significant differences between the genders, where men had a lower frequency than women (6 vs. 8 MN), and a higher nuclear index. In addition, age affected MN frequency; there was a positive correlation of MN frequency with age. Significant differences were found when comparing the moderate and highly exposed groups with the reference group.

In 2012, Zúñiga-Violante et al., determined the genetic damage among agricultural workers from Valle de San Quintín, Baja California, México [46]. The MN test, which blocks cytokinesis in peripheral blood samples, was used. Men environmentally exposed had less genetic damage than women with an MN means of 8.1 ± 1.83 and 13.1 ± 1.7, respectively, whereas occupational exposure affected both sexes, men with a mean of MN equal to 15.9 ± 2.9, and women with 18.12 ± 1.7. The time of exposure at work was shown to be directly related to the increased MN.

On the other hand, in addition to the use of lymphocytes, the number of publications that use oral mucosa cells with the MN test has increased greatly in recent decades, possibly due to its technical simplicity and the variety of complementary toxicological markers that can be used, as well as the fact that this method is inexpensive, and fast, with no need for establishing cell culture of three days.

These biomarkers have been widely used to determine the risk associated with exposure to pesticides. The analysis in epithelial cells is relevant because almost 92% of the different types of cancer are of epithelial origin and the epithelial cells of the mouth and nose are the first to have contact with pesticides after inhalation. Of course, there is also a study from Bonassi et al. (2011) who demonstrated a strong correlation between DNA damage found in lymphocytes as MN with MN frequency in epithelial buccal cells [126,127].

Gómez-Arroyo et al. (2000) evaluated cytogenetic damage in floriculturists of Morelos State, Mexico, exposed to pesticides from the greenhouses in towns of Santa Catarina, Jiutepec and Yecapixtla (application of chemicals to the flowers is uncontrolled there) and compared the results to the non-exposed group (people from the town of Temisco- their activity was not related to pesticides) [34]. Significant differences were found for mitotic index, cell proliferation kinetics and in MN frequency with the MN values being three times higher than in the non-exposed group. This analysis shows that exposure to pesticides significantly increases genetic damage, which implies that this tissue is altered at the chromosomal level and that it undergoes chromosome disruption and/or alterations of the mitotic spindle, together with other NAs.

Carbajal-López et al. (2016), evaluated the genotoxic effect of pesticides in exfoliated buccal cells of workers occupationally exposed in the state of Guerrero in the Tierra Caliente region [53]. With only male participants, 111 agricultural workers in three rural communities (Arcelia 62, Ajuchitlan 13, and Tlapehuala 36) and a control group of 60 individuals were evaluated to determine the effect produced by exposure to pesticides using the MN test on oral mucosal cells. The difference between the MN frequencies of the exposed and non-exposed populations was statistically significant. The same occurred for binucleated cells in the three localities without presenting a correlation of MN frequency with the exposure time. Picnosis and nuclear buds were observed in Arcelia and Tlapehuala; condensed chromatin was recorded in Arcelia and Ajuchitlán. The consumption of alcohol, age and smoking habit did not influence the study results [53]. Farmworkers in Maneadero, Baja California, constantly exposed to mixtures of pesticides and a group of non-exposed women from Ensenada, were evaluated together with their children to determine the genotoxic effects in cells of the oral mucosa [54]. The cell frequencies with MN and NA presented significant differences between the agricultural workers and the non-exposed group. The same occurred with the samples of the children, demonstrating that both direct exposure (agricultural workers) and indirect exposure (children of agricultural workers) were at risk of genotoxic damage. Frequencies of MN and NA in 2000 cells were obtained from the buccal mucosa from 144 individuals divided into four groups: (1) farmers (*n* = 37), (2) unexposed (*n* = 35), (3) farmers’ children (*n* = 34), and (4) unexposed children (*n* = 38). Differences were found between farmers and unexposed women in MN (*p* < 0.0001), condensed chromatin (*p* = 0.3376), and pyknotic cell frequency (*p* < 0.0001). Exposed children demonstrated higher significant frequencies in MN (*p* < 0.0001), lobulated nuclei (*p* < 0.0001), condensed chromatin (*p* < 0.0001), and pycnotic cell frequency (*p* < 0.004) when compared to unexposed children. The genotoxic effects of pesticides on workers occupationally exposed to these compounds during their aerial application in agricultural fields in Sinaloa were evaluated in the study of Martínez-Valenzuela et al. (2017) [56]. The cohort study involved 30 pilots of small airplanes who worked as aerial pesticide applicators and 30 non-exposed individuals as a control group. The MN test and the induction of other NA in oral mucosal epithelial cells were used to evaluate the genotoxic damage. The highest frequency ratios (FR) equal to 269.5 corresponded to binucleated cells followed by 54.2, corresponding to cells with pyknotic nuclei, 45.2 of cells with chromatin condensation, 3.7 of cells with broken-egg, 3.6 of cells with MN, and 2.0 of karyolitic cells. Age, time worked, smoking, and alcohol consumption did not have a significant influence on nuclear abnormalities in the pilots studied. Pesticide exposure was the main factor for nuclear abnormality results and DNA damage. Marked genotoxic damage was developed in even younger pilots with 2 years of work experience, caused by their daily occupational exposure to pesticides. As in other studies, the damages found were not influenced by smoking habits or the consumption of alcoholic beverages. Sánchez-Alarcón et al. (2016a) compared 32 agricultural workers in the rural community of Tlaxcala, Mexico, with 30 nonexposed individuals and also found elevated DNA buccal damage, but not connected with age, gender or smoking status [52]. Ortega-Martínez et al. (2014, 2019) compared 40 non exposed and 40 workers in high-tech and low-tech greenhouses of Atlixco City, in Puebla, Mexico, and demonstrated statistically higher values of NA in greenhouses workers, with frequency index ratios of 63.0 binucleated cells, 14.2 cells with condensed chromatin, 8.0 karyolytic cells, 3.8 cells pycnotic, 2.4 cells with nuclear outbreaks, and 2.3 cells with MN [62,128]. The highest NA frequency was found in workers in low-tech greenhouses compared to high-tech greenhouses. The male population of low-tech greenhouses showed higher frequencies of pycnotic cells, MN, and cells with nuclear outbreaks. Martínez-Valenzuela et al. (2009) evaluated genotoxic damage in 70 agricultural workers, 25 women and 45 men, exposed to pesticides in Las Grullas, Ahome, Sinaloa, Mexico, with an average of 7 years of exposure, and compared the results with non-exposed group consisting of 70 other persons, 21 women and 47 men from the city of Los Mochis, Sinaloa, Mexico [43].

The Mann–Whitney U-test showed significant differences in cellular proliferation kinetics (replication index and mitotic index evaluated together), MN and NA frequencies. On the other hand, they found no correlation between the registered MN frequencies, age, gender, exposure time, or any relationship with smoking habits and drinking alcoholic beverages.

Ruiz-Gamboa et al. (2016) compared 27 indoor sprayers exposed to pesticides with 26 non-exposed men and found a significantly higher MN frequency in sprayers 0.71/1000 cells (0.50–1.01) vs. controls 0.46/1000 cells (0.33–0.65) [55]. All NA: nuclear bud, binucleated, karyorrhexis, pyknosis, karyolysis, condensed chromatin were different between groups, and some workers had scores above regard: binucleated, pyknosis and karyorrhexis. Lazalde-Ramos et al. (2017), examined 120 of Mexico’s Indigenous individuals, including thirty from the ethnicities Cora, Huichol, Tarahumara and Tepehuano. Tepehuano and Tarahumaras showed the greatest damage to DNA, in both MN and NA, with a significant difference from the rest of the studied groups [57]. They also presented the group with the highest herbicide exposure (46.7%). In relation to smoking and drinking habits, these were more frequent in the Tarahumara group (33.3 and 50%).

Gómez-Arroyo et al. (2013) assessed the genotoxic risk for Mexican children who lived near an agricultural area with intense aerial pesticide applications. A significant increment of MN frequencies was observed [48]. In the same way, other nuclear abnormalities such as binucleated cells, nuclear buds, karyorrhexis and karyolysis also were detected; in all cases, the differences were significant in relation with the control group, indicating high health risks to the exposed children. However, in the genotoxic study carried out with the buccal cells of children from two elementary schools, one exposed to fumigations in agricultural fields, the other unexposed, no significant differences were found for the presence of MN, condensed chromatin and karyorrhexis between both groups of schoolchildren. Nevertheless, the most prevalent nuclear abnormalities in the exposed population were lobed nuclei, binucleated cells, apoptosis, and karyolysis, which showed significant differences between the unexposed and exposed schools [63].

#### 3.3.4. Alkaline Comet Assay

The alkaline comet assay, also called single cell gel electrophoresis (SCGE), is an electrophoretic technique for direct visualization of DNA damage in individual cells. In this technique, the cells (any type of the living cell, proliferating or non-proliferating, important for this technique is that cells are in single-cell suspension) are embedded in agarose gel on the slide. The cells are lysed by detergents and denaturated by alkaline solutions, then subjected to electrophoresis. Due to the size of the agarosis pores, free DNA (nucleus), if undamaged cannot move during the electrophoresis, while the damaged parts of the damaged DNA can migrate. Cells with more DNA damage show a greater migration of their DNA from the nucleus to the anode, giving the appearance of a comet (that is how the technique got its name, while the undamaged nuclei remain in the oval-round shape without the tail). Comets are observed under fluorescence microscopy after staining with suitable dyes such as ethidium bromide, acridine orange or iodinated propidium [129].

This method allows different types of DNA damage to be measured in human cells such as lymphocytes and has become an important tool in population assessment studies environmentally and/or occupationally exposed to different genotoxic agents, including radiation, chemical agents (pesticides, metals, air pollutants) and oxidative stress [130,131,132]. It stands out for its versatility and its potential applications. In terms of its simplicity, cost, the requirement of small cell samples, sensitivity and reliability, the comet assay and its different modifications have few serious competitors. This assay can provide invaluable information in the area of risk assessment of environmental and/or occupational exposures, as well as diseases related to oxidative stress and investigation of individual variation in relation to the response to DNA damage that may reflect genetic and/or environmental influences [133,134].

Using neutral conditions for lysis and electrophoresis, double-strand breaks can be detected. The alkaline version is able to detect, in addition to double chain breaks, single-chain breaks, alkali sensitive sites, DNA/DNA crosslinks or DNA-proteins associated with repair sites by incomplete excision in individual cells [122]. Due to inexpensive, easy and quick performance, the comet assay is used worldwide [135].

Using the comet assay, Yáñez et al. (2004), show that, chronic exposure to DDT induced DNA damage in women from malarious communities, a significant correlation was found between DNA damage and concentrations of DDT, DDD, and DDE, in blood [36]. However, Ortiz-Pérez et al. (2005), assessing DNA damage in children before and 24 h after indoor spraying of deltamethrin in malarious areas, found no significant differences in comet assay parameters [38].

Castillo-Cadena et al. (2006), determined the values of DNA damage in Mexican flower growers exposed to mixtures of pesticides for several years by determining the length/width index (T/N index), which they obtained by dividing the length of the tail of the comet between the diameter of the head of the nucleus [40]. The values found for the T/N index were 1.29, 1.40 and 1.67, for the control, environmentally exposed and directly exposed to pesticides groups, respectively. When applying the corresponding statistical tests, they found differences between the control group and the exposed group. As for confounding variables, such as gender, age, smoking habits, ingestion of alcoholic beverages, as well as exposure time (years), they determined that only smoking and drinking habits had an impact on the damages found.

After obtaining samples of maternal and umbilical cord blood, fifty mother-child pairs from an agricultural community in San Luis Potosí were studied, with the purpose of evaluating the exposure to mixtures of organochlorine pesticides and determining genotoxic damage by obtaining samples of maternal blood and the umbilical cord [44]. The comet test was evaluated using the intensity of the fluorescence and the length of the cauda or “olive tail moment”, considering a value of 4 as a reference, finding that 60% of the mothers and 78% of the babies, showed damage genotoxic with statistical significance. They also found 15 pesticides in maternal as well as in cord plasma.

Carbajal-López et al. (2016) also analyzed the genotoxic effect produced by exposure to mixtures of pesticides using the comet assay in exfoliated cells of the oral mucosa of 111 agricultural workers occupationally exposed in the state of Guerrero in three rural communities (Arcelia 62, Ajuchitlan 13, and Tlapehuala 36), and compared it with 60 non-exposed individuals [53]. Comet tail length was evaluated in 100 nuclei. Significant differences were found when the three exposed groups (Arcelia, Ajuchitlan and Tlapehuala) were compared with the non-exposed group in tail migration of DNA, with no positive correlation between exposure time and tail length and no significant effect of age, smoking, and alcohol consumption. In the study of Zepeda-Arce et al. (2017), 208 participants were divided into three groups according to their degree of exposure to pesticides: 22 individuals as the reference group not exposed to pesticides, 126 individuals moderately exposed and 60 people with high exposure, with the purpose of determining the magnitude of genetic damage induced by exposure, determined the tail- and Olive-tail moment in human lymphocytes [58]. The results showed an increase in the parameters suggesting genetic damage, together with the effect of pesticide-tobacco co-exposure (smoking habit), and the co-effect was more pronounced among women.

The comet assay can also be done on buccal cells with a somewhat different protocol. There is a review comparing different protocols and finding the best one for buccal comet assays [136]. As we explained, buccal cell samples are much easier to obtain and less invasive than blood sampling. Vazquez Boucard et al. (2017), demonstrated with the comet assay in 107 male buccal samples that people who drank well water or tap water rich in organochlorine pesticides and heavy metals detected in Todos Santos, BCS, Mexico presented elevated levels of DNA damage [59].

Jasso-Pineda et al. (2015), demonstrated elevated DNA blood level damage in 276 children living in areas at high risk of contamination in Mexico in eleven communities in four states of Mexico [51]. Children exposed to a chemical mixture among them polycyclic aromatic hydrocarbons (PAHs) and also DDT that was also detected in their urine samples had the significantly highest DNA damage level (*p* < 0.05) in their blood cells (olive tail moment = 7.5 ± 3.5), when compared with DNA damage levels in children living in the other scenarios assessed in this work.

To assess the genotoxic effects of pesticides in pilots occupationally exposed to these chemicals during aerial application in agricultural fields, the alkaline comet assay was performed on freshly collected peripheral whole blood lymphocytes from 30 pilots who applied aerial pesticides and 30 controls. The median of comet frequency, tail length and tail moment revealed statistically significant differences between the exposed subjects and controls [61].

### 3.4. Other Biomarkers of the Effect

#### 3.4.1. Biomarkers of Oxidative Stress

Other biomarkers of effect are markers of oxidative damage. An increase in oxidative stress has been associated with a variety of adverse outcomes (and different disease processes), but the explicit relationships have yet to be more clearly defined, leaving uncertainty in the use of oxidative stress biomarkers to predict the long-term effects at organism/population-level (EPA) [79]. Some pollutants, such as pesticides, are capable of generating oxidative stress, which triggers adaptive mechanisms through protection systems, usually quantified in plasma, such as: oxidized glutathione (GSSG)/reduced glutathione (GSH) ratio and the glutathione reductase (GR), catalase (CAT), superoxide dismutase (SOD) and peroxidase activities. Macromolecules that can be affected include lipids, proteins and nucleic acids.

Zepeda-Arce et al. (2017), determined the levels of malondialdehyde (MDA), SOD, CAT, glutathione peroxidase (GPx), GR and the relation of the levels of antioxidant enzymes with DNA damage among agricultural workers (sprinklers), occupationally exposed to pesticides [58]. The results showed a marginally significant decrease in SOD and CAT activities in the highly exposed group when compared with the control group. For MDA, significant differences were found among workers with greater seniority when compared with recently incorporated or temporary sprinklers. In the moderately exposed group, a positive correlation was found between MDA levels and GPx activity. In the highly exposed group, a negative correlation was observed between GR and CAT, as well as between MDA levels and GPx activities. On the other hand, they reported a positive correlation between DNA damage parameters and MDA levels. All this suggests an important role of antioxidant enzymes for the protection of DNA damage caused by occupational exposure to pesticides.

In a similar cross-sectional study in 201 individuals, Xotlanihua-Gervacio et al. (2018), presented a negative correlation between MN frequency and GPx activities. Individuals were assigned to pesticide spraying [60]. They found a marginal correlation between MN and glutathione reductase GR, as well as with superoxide dismutase SOD and found no correlation between MN and CAT. The geometric mean (GM) for the antioxidant enzymes was 198.68 U/mL for glutathione peroxidase GPx, 38.96 U/g Hb for GR, 94.78 U/mL for SOD, and 69.77 U/g Hb for CAT.

#### 3.4.2. Aneuploidies

Recio et al. (2001) evaluated the frequency of aneuploidies in sperm (X, Y, 18) in agricultural workers [35]. The samples were obtained before and during the pesticide application season. Aneuploidies were found in 0.67% of the total sperm nuclei; the most frequent was the absence of a sex chromosome or without sex chromosomes (0.19%), followed by XY18 (0.15%) and XY18–18 (0.06%).), finding a correlation between the concentration of the metabolites of OF pesticides and an increase in the frequency of aneuploid sperm. Thus, exposure to OFs can interfere with chromosomal segregation in sperm and increase the risk of genetic syndromes, such as Turner syndrome.

#### 3.4.3. Alterations in the Sperm Chromatin Structure

The analysis of the structure of the sperm chromatin (SCSA) is a relevant factor for reproductive toxicology since chromatins can be damaged at any time along the male reproductive tract, compromising fertility and development of offspring [37]. The SCSA measurements show that the structure of the sperm chromatin was altered in all the samples obtained from agricultural workers, finding that 75% of them were classified with poor fertility potential and only 12% were considered potentially fertile in comparison with the control population that presented only 4% with poor fertility potential.

Sperm quality has been negatively associated with the urinary level of dimethyldithiophosphate (DMDTP), a metabolite of OP in agricultural workers from Villa Juarez, Durango, Mexico [65]. Workers with carbofuran exposure at work for at least 3 years showed multinucleation of spermatozoa and spermatids [45]. Details of Mexican agricultural workers’ OP exposure associated with decreased semen quality, sperm DNA damage, etc., are further analysed in the review of Sánchez-Guerra et al., 2011 [100].

## 4. Conclusions

Humans are exposed to a large number of environmental hazards that can affect the functioning of specific biomolecules and thus affect health at different levels. Alterations to DNA are recognized as indicators of early damage in affected organisms; therefore, identifying the genotoxic potential of xenobiotics has been a beneficial and effective strategy in risk assessment.

Biomarkers allow one to improve the process of evaluation of health risks due to exposure to environmental xenobiotics. They can be used to calculate the exposure and the internal dose received by individuals and groups, with the consequent identification of those who suffer a greater or lesser risk than average.

New biomarkers, such as miRNAs, must be validated before applying them to risk assessments, which means that the relationship between the biomarker, the exposure and the effect or damage to health must be determined. The process of selecting and validating biomarkers for monitoring and evaluation requires careful consideration of the relevance and accuracy of the tests. The extent to which individuals are at lower or higher risk depends on the strength of the relationship between the positive result and the risk of developing effects on health.

Finding answers to many of the key challenges will continue to be an important factor in the investigation of the role, if any, of these compounds in the transmission of human diseases. These challenges include an accurate assessment of the role of exposure to pesticides in the multifactorial cause of the disease, latency, publication of negative results, classification errors, low-level dose measurement, accurate diagnosis, the role of biomarkers, adequate study design and adequate foundation.

The expansion of the repertoire of biomarkers available for exposure to pesticides and the use of several well-designed study protocols will provide critical tools in assessing the safety of pesticides and designing appropriate measures to minimize adverse exposures. Thus, the combination of in vitro data in animals and in humans will provide the best picture of the performance of a marker.

Occupational exposure to pesticide mixtures has been associated with an increase in genotoxic damage. This seems to depend on the degree and time of exposure as well as the hereditary characteristics of individuals (polymorphic genes involved in the metabolism of chemical agents and DNA repair mechanisms). Because of this, it is difficult to attribute the genetic damage found in agricultural workers to any particular pesticide.

Although new products have been developed, from organochlorine compounds, which have been replaced by OP and carbamates, and more recently by pyrethroids, which represent the class of pesticide compounds most frequently used today, different biomarkers can be used in monitoring studies to determine the risk associated with exposure to pesticides. It should be mentioned that “exposure to pesticides” is a very broad term that involves complex mixtures of compounds and many variables that can reduce or enhance their risks, especially in occupationally exposed groups.

As seen in this review, there are few studies conducted in Mexico where the risk of exposure to pesticides in occupationally exposed populations was evaluated. We should mention the valuable study and review of Sánchez-Guerra et al. from 2011 about epidemiological and experimental studies in Mexico on OP, but not all biomarkers were included in this review, and they were considered only regarding OP [100]. Further, it should be considered that workers usually are not exposed only to a particular product or group of pesticides, but to mixtures whose composition is often unknown to the workers until the time for sprinkling, or when those chemicals are measured in their samples.

On the other hand, there is no consistency in response to these types of exposures, either due to poor definition of exposure (according to Sivério et al., 2017), or to the different design of the studies, including the small number of participants [137]. Besides the problem of the sample size, there are differences in the geographic and meteorological characteristics of the agricultural areas and regions directly affecting exposure and influencing the results. These differences refer to the pesticide types, to the frequency of use, to the difference in exposure levels and duration, to the hours per day and years of working with the pesticides, and to the use of protective equipment. Results from our review support the use of miRNAs as biomarkers of exposure to pesticides, especially for their potential to understand the possible mechanisms of action of pesticides.

## Figures and Tables

**Table 1:** Studies developed in Mexico on genotoxicity biomarkers in populations exposed to pesticides.

Year of Study/Location (State)	Genotoxic Endpoint/Bioassay	No. Exposed/No. Controls	Biomarkers and Exposure Correlation	Authors
1987/ND	CA/PBL	26/26	Positive *p* < 0.01	Zapata-Gayón et al. [31]
1992/Tlaxcala	SCE/PBL	94/76	Negative *p* = 0.4	Gómez-Arroyo et al. [32]
1997/Morelos	SCE/Blood samples	49/31	Positive *p* = 0.03	Steenland et al. [33]
	CA/Blood samples		Positive *p* = 0.05	
2000/Morelos	SCE/PBL	30/30	Positive *p* < 0.001	Gómez-Arroyo et al. [34]
	MN/exfoliated buccal mucosa cells			
2001/Durango	Sex null aneuploidy/Sperm	9/9	Negative *p* < 0.07	Recio et al. [35]
2004/San Luis Potosi	Comet assay/PBMC	Total 54	Positive *p* < 0.05	Yañez et al. [36]
2004/Durango	Chromatin structure/Sperm	Total 33	Positive *p* < 0.05	Sánchez-Peña et al. [37]
2005/San Luis Potosi	Comet assay/PBMC	Total 28	Negative	Ortiz-Pérez et al. [38]
2005/Chihuahua&Mexico City	MN/umbilical cord lymphocytes	21/16	Positive *p* < 0.01	Levario-Carrillo et al. [39]
2006/Mexico	Comet assay/leukocytes	52/38	Positive *p* < 0.001	Castillo-Cadena et al. [40]
2006/Tlaxcala	MN/peripheral lymphocytes	28/44	Negative *p* > 0.05	Montero et al., 2006 [41]
2008/Yucatan	DNA integrity/sperm	Total 54	Positive *p* < 0.05	Pérez-Herrera et al. [42]
2009/Sinaloa	SCE/PBL	70/70	Positive *p* < 0.001	Martínez-Valenzuela et al. [43]
	MN and NA/exfoliated buccal cells			
2009/San Luis Potosi	Comet assay/blood samples	Total 50	Negative	Alvarado et al. [44]
	MN/blood samples			
2010/Nuevo Leon	Multinucleation/Sperm	Total 2	Positive	Gallegos-Avila et al. [45]
2012/Baja California	CBMN/peripheral blood samples	25/15	Positive *p* < 0.05	Zúñiga-Violante et al. [46]
2012/Jalisco	DNA fragment quantification/blood	25/21	Positive *p* = 0.00006	Payán-Rentería et al. [47]
2013/Sinaloa	MN and NA/exfoliated buccal cells	125/125	Positive *p* < 0.001	Gómez-Arroyo et al. [48]
2013/San Luis Potosi	Comet assay/blood simples	Total 50	Negative	Alvarado-Hernández et al. [49]
	MN/blood samples			
2014/Baja California	CBMN/PBL	26/22	Positive *p* < 0.05	Montaño-Soto et al. [50]
2015/Chiapas	Comet assay/Blood cells	35/25	Positive *p* < 0.05	Jasso-Pineda et al. [51]
2015/Oaxaca		20/25		
2015/Quintana Roo		21/25		
2016/Tlaxcala	MN and NA/mucosa buccal cells	32/30	Positive	Sánchez-Alarcón et al. [52]
2016/Guerrero	Comet assay/exfoliated buccal cells	111/60	Positive *p* < 0.001	Carbajal-López et al. [53]
	MN and NA/exfoliated buccal cells			
2016/Baja California	MN and NA/buccal cells	71/73	Positive *p* < 0.0001	Castañeda-Yslas et al. [54]
2016/Yucatan	MN and NA/buccal mucosa cells	27/26	Positive *p* < 0.001	Ruiz-Gamboa et al. [55]
2017/Sinaloa	MN and NA epithelial oral mucosa cells	30/30	Positive *p* < 0.05	Martínez-Valenzuela et al. [56]
2017/Durango	MN and NA/oral mucosa	30/30	Positive *p* < 0.05	Lazalde-Ramos et al. [57]
2017/Nayarit	Comet assay/whole blood	60/22	Negative	Zepeda-Arce et al. [58]
2017/Baja California	Comet assay/buccal cells	57/24	Positive *p* < 0.0001	Vazquez Boucard et al. [59]
2018/Nayarit	CBMN/whole blood	Total 201	Negative	Xotlanihua-Gervacio et al. [60]
2018/Sinaloa	Comet assay/PBL	30/30	Positive *p* < 0.05	Martínez-Valenzuela et al. [61]
2019/Puebla	MN and NA/oral mucosa	40/40	Positive *p* < 0.05	Ortega-Martínez et al. [62]
2020/Baja California	MN and NA/buccal cells	63/24	Positive *p* < 0.001	Anguiano-Vega et al. [63]

PBL—peripheral whole blood lymphocytes; PBMC—peripheral blood mononuclear cells.

**Table 2:** Studies developed in Mexico on biomarkers susceptibility in populations exposed to pesticides.

Year of Study/Location (State)	Biomarker	Exposed/Controls (*n*)	Result	Authors
1997/Morelos	TSH serum levels	49/31	Increment in TSH *p* = 0.05	Steenland et al. [33]
2005/Mexico City	Genetic PON1 polymorphism	Total 214	Frequencies of polymorphism PON1 -162A (0.21), -108C (0.45), 55L (0.84) and 192R (0.49)	Rojas-García et al. [64]
2008/Durango	Sperm quality	Total 52	OP exposure decreases sperm quality *p* < 0.05	Recio-Vega et al. [65]
2008/Yucatan	Susceptibility to OP toxicity and PON1Q192R	Total 54	Farmers with 192RR genotype were more susceptible	Pérez-Herrera et al. [42]
2009/Mexico	PON1 Q192R polymorphisms	Total 264	Correlation of risk having a baby with LBW and PON1 192RR genotype in floriculture mother	Moreno Banda et al. [66]
2009/Morelos				
2009/Mexico	PON1 polymorphisms	Total 170	Significant association between the PON1_192_ polymorphism and PON1 activity towards paraoxon and diazoxon	López-Flores et al. [67]
2009/Morelos				
2010/Mexico	TSH serum levels	Total 136	Increment in TSH *p* = 0.001	Lacasaña et al. [68]
2010/Morelos				
2010/Mexico	Tyroid function	Total 136	Association of OP and thyroid function in individuals with lower PON1 activity.	Lacasaña et al. [69]
2010/Morelos				
2010/Yucatan	PON1 polymorphisms	152/160	PON1 polymorphisms- relevant risk factors for offspring affected with SB	González-Herrera et al. [70]
2013/Mexico	PON1 polymorphisms	Total 264	maternal PON1 polymorphisms effect on miscarriage in exposed women	Blanco-Muñoz et al. [71]
2013/Morelos				
2016/Mexico	Thyroid hormones	Total 136	Positive associations: serum pesticides levels and thyroid hormones	Blanco-Muñoz et al. [72]
2016/Morelos				
2018/Nayarit	Antioxidante enzyme activities	Total 201	Glutathione peroxidase is involved in pesticide damage	Xotlanihua-Gervacio et al. [60]
2018/Yucatán	BuChE determination	27/26	No significant differences	Ruiz-Gamboa et al. [73]
2018/Sinaloa	Enzymatic activities	Total 49	Enzymatic activities altered by pesticides exposure	Galindo-Reyes and Alegria [74]
2019/Sonora	AChE activity	25/5	significant differences between exposed and control	Alvarado-Ibarra et al. [75]
2019/Mexico	PON1 polymorphisms	Total 381	Genotypes marginally associated with hypothyroxinemia	Torres Sánchez et al. [76]

OP—organophosphate pesticides.

## Data Availability

Not applicable.

## References

[1] Bolognesi C. (2003). Genotoxicity of pesticides: A review of human biomonitoring studies. Mutat. Res..

[2] Ali U., Syed J.H., Malik R.N., Katsoyiannis A., Li J., Zhang G., Jones K.C. (2014). Organochlorine pesticides (OCPs) in South Asian region: A review. Sci. Total Environ..

[3] Costa C., Teixeira J.P., Silva S., Roma-Torres J., Coelho P., Gaspar J., Alves M., Laffon B., Rueff J., Mayan O. (2006). Cytogenetic and molecular biomonitoring of a Portuguese population exposed to pesticides. Mutagenesis.

[4] Eastmond D.A., Balakrishnan S., Krieger R. (2010). Genotoxicity of Pesticides. Hayes’ Handbook of Pesticide Toxicology.

[5] Bolognesi C., Creus A., Ostroski-Wegman P., Marcos R. (2011). Micronuclei and pesticide exposure. Mutagenesis.

[6] WHO, IARC (2014). IARC Monographs on the Evaluation of Carcinogenic Risks to Humans.

[7] Polanco Rodríguez A.G., Navarro Alberto J.A., Solorio Sanchez J., Mena Rejon G.J., Marrufo Gómez J.M., DelValls Casillas T.A. (2015). Contamination by organo-chlorine pesticides in the aquifer of the ring of Cenotes in Yucatan, Mexico. Water Environ. J..

[8] Carvalho F.P. (2006). Agriculture, pesticides, food security and food safety. Environ. Sci. Policy.

[9] Van Maele-Fabry G., Libotte V., Willems J., Lison D. (2006). Review and meta-analysis of risk estimates for prostate cancer in pesticide manufacturing workers. Cancer Cause Control.

[10] Mathur V., John P.J., Soni I., Bhatnagar P. (2008). Blood levels of organochlorine pesticide residues and risk of reproductive tract cancer among women from Jaipur, India. Adv. Exp. Med. Biol..

[11] Khan Y., Khan T. (2013). Banned pesticides residues in farm gate vegetables of Ganganagar. Int. J. Sci. Nat..

[12] Mostafalou S., Abdollahi M. (2013). Pesticides and human chronic diseases: Evidences, mechanisms, and perspectives. Toxicol. Appl. Pharm..

[13] Sachin K., Rashmi S., Manish S., Siddhartha W., Uday L. (2013). Haemangiomas and venous malformations of the head and neck: A retrospective analysis of endovascular management in 358 patients. Indian J. Plast. Surg..

[14] Ruiz-Suarez L.E., Castro-Chan R.A., Rivero-Perez N.E., Trejo-Acevedo A., Guillen-Navarro G.K., Geissen V., Bello-Mendoza R. (2014). Levels of organochlorine pesticides in blood plasma from residents of malaria-endemic communities in Chiapas, Mexico. Int. J. Environ. Res. Public Health.

[15] Sultana J., Syed J.H., Mahmood A., Ali U., Rehman M.Y.A., Malik R.N., Li J., Zhang G. (2014). Investigation of organochlorine pesticides from the Indus Basin, Pakistan: Sources, airesoil exchange fluxes and risk assessment. Sci. Total Environ..

[16] Velasco A., Hernández S., Ramírez M., Ortíz I. (2014). Detection of residual organochlorine and organophosphorus pesticides in agricultural soil in Rio Verde region of San Luis Potosí, Mexico. J. Environ. Sci. Health B.

[17] USEPA (2017). Pesticided Worker Safety. Agricultural Worker Protection Standard (WPS). https://www.epa.gov/pesticide-worker-safety/agricultural-worker-protection-standard-wps.

[18] EFSA (European Food Safety Authority) (2019). Scientific report on the 2017 European Union report on pesticide residues in food. EFSA J..

[19] FAO, WHO (2016). Manual on Development and Use of FAO and WHO Specifications for Pesticides.

[20] FAO, WHO (2007). FAO/WHO Framework for the Provision of Scientific Advice on Food Safety and Nutrition.

[21] WHO—World Health Organization (1992). Our Planet, Our Health.

[22] Rodríguez Á.G.P., López M.I.R., DelValls Casillas T.Á., León J.A., Mahjoub O., Prusty A.K. (2017). Monitoring of organochlorine pesticides in blood of women with uterine cervix cancer. Environ. Pollut..

[23] Rodríguez-Pimentel L., Wilkins-Gamiz A., Olvera-Santamaría R., Silva-Romo R. (2005). Panorama epidemiologico de las intoxicaciones en México. Med. Interna Mex..

[24] INEGI (2010). Instituto Nacional de Estadística, Geografía e Informatica Continuo Nacional de la Carta Geologica del Estado de Yucatan.

[25] Bauer G.P., Gondwe B., Charvet G., Marín L., Rebolledo-Vieyra M., Merediz-Alonso G. (2011). Review: The Yucatan peninsula karst aquifer, Mexico. Hydrogeol. J..

[26] Barquera S., Campos-Nonato I., Hernández-Barrera L., Flores M., Durazo-Arvizu R., Kanter R., Rivera J.A. (2009). Obesity and central adiposity in Mexican adults: Results from the Mexican national health and nutrition survey 2006. Salud Pública Mex..

[27] Barquera S., Campos-Nonato I., Hernández-Barrera L., Pedroza-Tobías A., Rivera-Dommarco J.A. (2013). Prevalencia de obesidad en adultos mexicanos, ENSANUT 2012. Salud Pública Mex..

[28] Rodas-Ortíz J.P., Ceja-Moreno V., González-Navarrete R.L., Alvarado-Mejía J., Rodríguez-Hernández M.E., Gold-Bouchot G. (2008). Organochlorine pesticides, and polychlorinated biphenyls levels in human milk from Chelem, Yucatan, Mexico. Bull. Environ. Contam. Toxicol..

[29] Martínez-Salinas R.I., Díaz-Barriga F., Batres-Esquivel L.E., Pérez-Maldonado I.N. (2011). Assessment of the levels of DDT and its metabolites in soil and dust samples from Chiapas, Mexico. Bull. Environ. Contam. Toxicol..

[30] Rodríguez Á.G.P., López M.I.R., DelValls Casillas T.A., León J.A., Prusty B.A.K., Cervera F.J.Á. (2017). Levels of persistent organic pollutants in breast milk of Maya women in Yucatan, Mexico. Environ. Monit. Assess..

[31] Zapata Gayón N., Zapata Gayón C., González Angulo A. (1987). Clastogenic changes in the chromosomes of a population of individuals occupationally exposed to different pesticides. Salud Pública Mex..

[32] Gómez-Arroyo S., Noriega-Aldana N., Osorio A., Galicia F., Ling S., Villalobos-Pietrini R. (1992). Sister-chromatid Exchange analysis in a rural population of Mexico exposed to pesticides. Mutat. Res..

[33] Steenland K., Cedillo L., Tucker J., Hines C., Sorensen K., Deddens J., Cruz V. (1997). Thyroid hormones and cytogenetic out-comes in backpack sprayers using ethylenebis (dithiocarbamate) (EBCD) fungicides in Mexico. Environ. Health Perspect..

[34] Gómez-Arroyo S., Díaz-Sánchez Y., Meneses-Pérez M.A., Villalobos-Pietrini R., De León-Rodríguez J. (2000). Cytogenetic bio-monitoring in a Mexican floriculture worker group exposed to pesticides. Mutat. Res..

[35] Recio R., Robbins W.A., Borja-Aburto V., Morán-Martínez J., Froines J.R., Hernández R.M., Cebrián M.E. (2001). Organophos-phorous pesticide exposure increases the frequency of sperm sex null aneuploidy. Environ. Health Perspect..

[36] Yáñez L., Borja-Aburto V.H., Rojas E., de la Fuente H., González-Amaro R., Gómez H., Jongitud A.A., Díaz-Barriga F. (2004). DDT induces DNA damage in blood cells. Studies in vitro and in women chronically exposed to this insecticide. Environ. Res..

[37] Sánchez-Peña L.C., Reyes B.E., López-Carrillo L., Recio R., Morán-Martínez J., Cebrián M.E., Quintanilla-Vega B. (2004). Or-ganophosphorous pesticide exposure alters sperm chromatin structure in Mexican agricultural workers. Toxicol. Appl. Pharmacol..

[38] Ortiz-Pérez M.D., Torres-Dosal A., Batres L.E., López-Guzmán O.D., Grimaldo M., Carranza C., Pérez-Maldonado I.N., Martínez F., Pérez-Urizar J., Díaz-Barriga F. (2005). Environmental health assessment of deltamethrin in a malarious area of Mexico: Environmental persistence, toxicokinetics, and genotoxicity in exposed children. Environ. Health Perspect..

[39] Levario-Carrillo M., Sordo M., Rocha F., González-Horta C., Amato D., Ostrosky-Wegman P. (2005). Micronucleus frequency in human umbilical cord lymphocytes. Mutat. Res..

[40] Castillo-Cadena J., Tenorio-Vieyra L.E., Quintana-Carabia A.I., García-Fabila M.M., Ramírez-San Juan E., Madrigal-Bujaidar E. (2006). Determination of DNA damage in floriculturists exposed to mixtures of pesticides. J. Biomed. Biotechnol..

[41] Montero R., Serrano L., Araujo A., Dávila V., Ponce J., Camacho R., Morales E., Méndez A. (2006). Increased cytogenetic dam-age in a zone in transition from agricultural to industrial use: Comprehensive analysis of the micronucleus test in peripheral blood lymphocytes. Mutagenesis.

[42] Pérez-Herrera N., Polanco-Minaya H., Salazar-Arredondo E., Solís-Heredia M.J., Hernández-Ochoa I., Rojas-García E., Alvarado-Mejía J., Borja-Aburto V.H., Quintanilla-Vega B. (2008). PON1Q192R genetic polymorphism modifies organophosphorous pesticide effects on semen quality and DNA integrity in agricultural workers from southern Mexico. Toxicol. Appl. Pharmacol..

[43] Martínez-Valenzuela C., Gómez-Arroyo S., Villalobos-Pietrini R., Waliszewski S., Calderón-Segura M.E., Félix-Gastélum R., Álvarez-Torre A. (2009). Genotoxic biomonitoring of agricultural workers exposed to pesticides in the north of Sinaloa State, Mexico. Environ. Int..

[44] Alvarado D., Yáñez L., Montero R. (2009). Organochlorine pesticides mixture exposure assessment and DNA damage in moth-er-child pairs in agricultural community in San Luis Potosi, Mexico. Toxicol. Lett..

[45] Gallegos-Avila G., Ancer-Rodríguez J., Niderhauser-García A., Ortega-Martínez M., Jaramillo-Rangel G. (2010). Multinucleation of spermatozoa and spermatids in infertile men chronically exposed to carbofuran. Reprod. Toxicol..

[46] Zúñiga-Violante E., Arellano García E., Ojinaga L.C., Heusser W.D., Von-Glascoe C., Aguilera J.C.L., Ruiz B. (2012). Daño genético y exposición a plaguicidas en trabajadores agrícolas del Valle de San Quintín, Baja California, México. Rev. Salud Ambient..

[47] Payán-Rentería R., Garibay-Chávez G., Rangel-Ascencio R., Preciado-Martínez V., Muñoz-Islas L., Beltrán-Miranda C., Mena-Munguía S., Jave-Suárez L., Feria-Velasco A., De Celis R. (2012). Effect of chronic pesticide exposure in farm workers of a Mexico community. Arch. Environ. Occup Health.

[48] Gomez-Arroyo S., Martinez-Valenzuela C., Calvo-Gonzalez S., Villalobos-Pietrini R., Waliszewski S.M., Calderón-Segura M.E., Martínez-Arroyo S., Félix-Gastelum R., Lagarda-Escarrega A. (2013). Assessing the genotoxic risk for mexican children who are in residential proximity to agricultural areas with intense aerial pesticide applications. Rev. Int. Contam. Ambient..

[49] Alvarado-Hernandez D.L., Montero-Montoya R., Serrano-Garcia L., Arellano-Aguilar O., Jasso-Pineda Y., Yáñez-Estrada L. (2013). Assessment of exposure to organochlorine pesticides and levels of DNA damage in mother-infant pairs of an agrarian community. Environ. Mol. Mutagenes..

[50] Montaño-Soto T., Arellano-García E., Camarena Ojinaga L., Vonglascoe C., Ruiz-Ruiz B. (2014). Genotoxic biomonitoring and exposure to pesticides in women laborers at Maneadero Valley in Baja California, Mexico. Int. J. Appl. Nat. Sci. (IJANS).

[51] Jasso-Pineda Y., Díaz-Barriga F., Yáñez-Estrada L., Pérez-Vázquez F.J., Pérez-Maldonado I.N. (2015). DNA damage in Mexican children living in high-risk contaminated scenarios. Sci. Total Environ..

[52] Sánchez-Alarcón J., Pérez-Zempoalteca Y., Milić M., Montiel-González J.M.R., Valencia-Sánchez R.A., Valencia-Quintana R. (2016). Analysis of micronucleus and nuclear abnormalities in mucosa buccal cells of agricultural workers exposed to pesticide mixtures in Tlaxcala state, Mexico. Toxicol. Lett..

[53] Carbajal-López Y., Gómez-Arroyo S., Villalobos-Pietrini R., Calderón-Segura M.E., Martínez-Arroyo A. (2016). Biomonitoring of agricultural workers exposed to pesticide mixtures in Guerrero state, Mexico, with comet assay and micronucleus test. Environ. Sci. Pollut. Res. Int..

[54] Castañeda-Yslas I.J., Arellano-García M.E., García-Zarate M.A., Ruíz-Ruíz B., Zavala-Cerna M.G., Torres-Bugarín O. (2016). Biomonitoring with micronuclei test in buccal cells of female farmers and children exposed to pesticides of Maneadero agricultural valley, Baja California, Mexico. J. Toxicol..

[55] Ruiz-Gamboa K., Pérez-Herrera N., Cámara-Vallejos R., Medina-Moreno M., Albertos-Alpuche N., Esperón-Hernández R., Zapata-Vázquez R., Rojas-García A., Medina-Díaz I. (2016). Genotoxic effect in exfoliated buccal cells from indoor sprayers exposed to pesticides in southern Mexico. Toxicol. Lett..

[56] Martínez-Valenzuela C., Waliszewski S.M., Amador-Muñoz O., Meza E., Calderón-Segura M.E., Zenteno E., Huicha-pan-Martínez J., Caba M., Félix-Gastélum R., Longoria-Espinoza R. (2017). Aerial pesticide application causes DNA damage in pilots from Sinaloa, Mexico. Environ. Sci. Pollut. Res. Int..

[57] Lazalde-Ramos B.P., Zamora-Pérez A.L., Sosa-Macías M., Galaviz-Hernández C., Zúñiga-González G.M. (2017). Micronuclei and nuclear anomalies in Mexico’s indigenous population. Salud Pública Mex..

[58] Zepeda-Arce R., Rojas-García A.E., Benitez-Trinidad A., Herrera-Moreno J.F., Medina-Díaz I.M., Barrón-Vivanco B.S., Villegas G.P., Hernández-Ochoa I., Sólis Heredia M.J., Bernal-Hernández Y.Y. (2017). Oxidative stress and genetic damage among workers exposed primarily to organophosphate and pyrethroid pesticides. Environ. Toxicol..

[59] Vazquez Boucard C., Lee-Cruz L., Mercier L., Ramírez Orozco M., Serrano Pinto V., Anguiano G., Cazares L., Díaz D. (2017). A study of DNA damage in buccal cells of consumers of well- and/or tap-water using the comet assay: Assessment of occupational exposure to genotoxicants. Environ. Mol. Mutagenes..

[60] Xotlanihua-Gervacio M.D.C., Guerrero-Flores M.C., Herrera-Moreno J.F., Medina-Díaz I.M., Bernal-Hernández Y.Y., Barrón-Vivanco B.S., Sordo M., Rojas-García A.E. (2018). Micronucleus frequency is correlated with antioxidant enzyme levels in workers occupationally exposed to pesticides. Environ. Sci. Pollut. Res. Int..

[61] Martínez-Valenzuela C., Waliszewski S., Calderón-Segura M.E., Caba M., Meza E., Gómez-Arroyo S., Amador-Muñoz O., Villalobos-Pietrini R., Huichapan Martínez J., Ortega-Martínez L.D. (2018). Comet Assay results of pilots exposed to pesticides. Acta Univ..

[62] Ortega-Martínez L.D., Pérez-Armendáriz B., Waliszewski S., Gómez-Arroyo S., Baños-Lara M.R., Terán-Cervantes M., Castro-Carranza G., Martínez-Valenzuela M.C. (2019). Daño genético y citotóxico provocado por plaguicidas en jornaleros que la-boran en invernaderos en Atlixco, Puebla, México (Genetic and cytotoxic damage induced by pesticides in greenhouse workers in Atlixco, Puebla, Mexico). Rev. Int. Contam. Ambient..

[63] Anguiano-Vega G.A., Cazares-Ramirez L.H., Rendon-Von Osten J., Santillan-Sidon A.P., Vazquez-Boucard C.G. (2020). Risk of genotoxic damage in schoolchildren exposed to organochloride pesticides. Sci. Rep..

[64] Rojas-García A.E., Solís-Heredia M.J., Piña-Guzmán B., Vega L., López-Carrillo L., Quintanilla-Vega B. (2005). Genetic polymor-phisms and activity of PON1 in a Mexican population. Toxicol. Appl. Pharmacol..

[65] Recio-Vega R., Ocampo-Gómez G., Borja-Aburto V.H., Moran-Martínez J., Cebrian-Garcia M.E. (2008). Organophosphorus pesti-cide exposure decreases sperm quality: Association between sperm parameters and urinary pesticide levels. J. Appl. Toxicol..

[66] Moreno-Banda G., Blanco-Muñoz J., Lacasaña M., Rothenberg S.J., Aguilar-Garduño C., Gamboa R., Pérez-Méndez O. (2009). Maternal exposure to floricultural work during pregnancy, PON1 Q192R polymorphisms and the risk of low birth weight. Sci. Total Environ..

[67] López-Flores I., Lacasaña M., Blanco-Muñoz J., Aguilar-Garduño C., Sanchez-Villegas P., Pérez-Méndez O.A., Gamboa-Avila R. (2009). Relationship between human paraoxonase-1 activity and PON1 polymorphisms in Mexican workers exposed to organophosphate pesticides. Toxicol. Lett..

[68] Lacasaña M., López-Flores I., Rodríguez-Barranco M., Aguilar-Garduño C., Blanco-Muñoz J., Pérez-Méndez O., Gamboa R., Bassol S., Cebrian M.E. (2010). Association between organophosphate pesticides exposure and thyroid hormones in floriculture workers. Toxicol. Appl. Pharmacol..

[69] Lacasaña M., López-Flores I., Rodríguez-Barranco M., Aguilar-Garduño C., Blanco-Muñoz J., Pérez-Méndez O., Gamboa R., Gonzalez-Alzaga B., Bassol S., Cebrian M.E. (2010). Interaction between organophosphate pesticide exposure and PON1 activity on thyroid function. Toxicol. Appl. Pharmacol..

[70] González-Herrera L., Martín Cerda-Flores R., Luna-Rivero M., Canto-Herrera J., Pinto-Escalante D., Perez-Herrera N., Quintanilla-Vega B. (2010). Paraoxonase 1 polymorphisms and haplotypes and the risk for having offspring affected with spina bifida in Southeast Mexico. Birth Defects Res. Part A Clin. Mol. Teratol..

[71] Blanco-Muñoz J., Aguilar-Garduño C., Gamboa-Avila R., Rodríguez-Barranco M., Pérez-Méndez O., Huesca-Gómez C., González-Alzaga B., Lacasaña M. (2013). Association between PON1 genetic polymorphisms and miscarriage in Mexican women exposed to pesticides. Sci. Total Environ..

[72] Blanco-Muñoz J., Lacasaña M., López-Flores I., Rodríguez-Barranco M., González-Alzaga B., Bassol S., Cebrian M.E., López-Carrillo L., Aguilar-Garduño C. (2016). Association between organochlorine pesticide exposure and thyroid hormones in floriculture workers. Environ. Res..

[73] Ruiz-Gamboa K., Cámara-Vallejo R., Medina-Moreno M., Albertos-Alpuche N., Esperón-Hernández R., Zapata-Vázquez R., Rojas A., Medina-Díaz M., Montero-Lara G., Moo-Huchin J. (2018). Occupational exposure to pesticides and knowledge about related policies in urban pest control operators from southeast Mexico. Rev. Int. Contam. Ambient..

[74] Galindo-Reyes J.G., Alegria H. (2018). Toxic effects of exposure to pesticides in farm workers in Navolato, Sinaloa (Mexico). Rev. Int. Contam. Ambient..

[75] Alvarado Ibarra J., Valencia López C.A., Castillo Moreno M.R., Luna Reyes P.D., Borboa Servin J.A., Mexia Apodaca M.E., Ruiz Sandoval N.C. (2019). Agroquímicos organofosforados y su potencial daño en la salud de trabajadores agrícolas del campo sonorense. CIENCIA Ergo-sum.

[76] Torres-Sánchez L., Gamboa R., Bassol-Mayagoitia S., Huesca-Gómez C., Nava M.P., Vázquez-Potisek J.I., Yáñez-Estrada L., Mejía-Saucedo R., Blanco-Muñoz J. (2019). Para-occupational exposure to pesticides, PON1 polymorphisms and hypothyrox-inemia during the first half of pregnancy in women living in a Mexican floricultural area. Environ. Health.

[77] Anwar W.A. (1996). Biomarkers of human exposure to pesticides. Environ. Health Perspect..

[78] Arango S.S. (2012). Biomarcadores para la evaluación de riesgo en la salud humana. Rev. Fac. Nac Salud Pública.

[79] EPA https://www.epa.gov/pesticide-science-and-assessing-pesticide-risks/defining-pesticide-biomarkers.

[80] Gil F. (2000). El papel de los biomarcadores en toxicología humana. Rev. Toxicol..

[81] Waliszewski S.M., Pardío V.T., Waliszewski K.N., Chantiri J.N., Aguirre A.A., Infanzón R.M., Rivera J. (1997). Organochlorine pesticide residues in cow’s milk and butter in Mexico. Sci. Total Environ..

[82] Waliszewski S.M., Aguirre A.A., Infanzon R.M., Silva C.S., Siliceo J. (2001). Organochlorine pesticide levels in maternal adipose tissue, maternal blood serum, umbilical blood serum, and milk from inhabitants of Veracruz, Mexico. Arch. Environ. Contam. Toxicol..

[83] Waliszewski S.M., Gómez-Arroyo S., Infanzón R.M., Villalobos-Pietrini R., Maxwell Hart M. (2003). Comparison of organochlorine pesticide levels between abdominal and breast adipose tissue. Bull. Environ. Contam. Toxicol..

[84] Waliszewski S.M., Bermúdez M.T., Infanzón R.M., Silva C.S., Carvajal O., Trujillo P., Gómez-Arroyo S., Villa-lobos-Pietrini R., Saldaña V.A., Melo G. (2005). Persistent organochlorine pesticide levels in breast adipose tissue in women with malignant and benign breast tumors. Bull. Environ. Contam. Toxicol..

[85] Waliszewski S.M., Valencia Quintana R., Corona C.A., Herrero M., Sánchez K., Aguirre H., Aldave I.A., Gómez Arroyo S., Villalobos Pietrini R. (2010). Comparison of organochlorine pesticide levels in human adipose tissue of inhabitants from Veracruz and Puebla, Mexico. Arch. Environ. Contam. Toxicol..

[86] Waliszewski S.M., Caba M., Herrero-Mercado M., Saldarriaga-Noreña H., Meza E., Zepeda R., Valencia Quintana R., Infanzon R. (2012). Organochlorine pesticide residue levels in blood serum of inhabitants from Veracruz, Mexico. Environ. Monit. Assess..

[87] Waliszewski S.M., Caba M., Saldarriaga-Noreña H., Martínez A.J., Meza E., Valencia Quintana R., Zepeda R. (2014). Organo-chlorine pesticide level differences among female inhabitants from Veracruz, Puebla and Tabasco, Mexico. Bull. Environ. Contam. Toxicol..

[88] Herrero-Mercado M., Waliszewski S.M., Caba M., Martínez-Valenzuela C., Hernández-Chalate F. (2010). Organochlorine pesti-cide levels in umbilical cord blood of newborn in Veracruz, Mexico. Bull. Environ. Contam. Toxicol..

[89] Herrero-Mercado M., Waliszewski S.M., Caba M., Martínez-Valenzuela C., Arroyo S.G., Villalobos Pietrini R., Cantú Martínez P.C., Hernández-Chalate F. (2011). Organochlorine pesticide gradient levels among maternal adipose tissue, maternal blood serum and umbilical blood serum. Bull. Environ. Contam. Toxicol..

[90] Gyalpo T., Fritsche L., Bouwman H., Bornman R., Scheringer M., Hungerbühler K. (2012). Estimation of human body concentra-tions of DDT from indoor residual spraying for malaria control. Environ. Pollut..

[91] Dirtu A.C., Dirinck E., Malarvannan G., Neels H., Van Gaal L., Jorens P.G., Covaci A. (2013). Dynamics of organohalogenated contaminants in human serum from obese individuals during one year of weight loss treatment. Environ. Sci. Technol..

[92] Calderón-Garcidueñas A.L., Waliszewski S.M., Ruiz-Ramos R., Del Carmen Martínez-Valenzuela M. (2018). Time trend tendency (1988–2014 years) of organochlorine pesticide levels in the adipose tissue of Veracruz inhabitants. Environ. Monit. Assess..

[93] Rodríguez Á.G.P., López M.I.R., DelValls Casillas Á., Araujo León J.A., datta Banik S. (2018). Impact of pesticides in karst groundwater. Review of recent trends in Yucatan, Mexico. Groundw. Sustain. Dev..

[94] López-Carrillo L., Torres-Arreola L., Torres-Sánchez L., Espinosa-Torres F., Jiménez C., Cebrián M., Waliszewski S., Sáldate O. (1996). Is DDT use a public health problem in Mexico?. Environ. Health Perspect..

[95] Katagi T. (2010). Bioconcentration, bioaccumulation and metabolism of pesticides in aquatic organisms. Rev. Environ. Contam. Toxicol..

[96] NACEC (2003). DDT No Longer Used in North America. http://www.cec.org/files/PDF/POLLUTANTS/DDT_en.pdf.

[97] NACEC (2006). The North American Regional Action Plan (NARAP) on Lindane and Other Hexachlorocyclohexane (HCH) Isomers.

[98] Li Y.F., Macdonald R.W. (2005). Sources and pathways of selected organochlorine pesticides to the Arctic and the effect of pathway divergence on HCH trends in biota: A review. Sci. Total Environ..

[99] Colović M.B., Krstić D.Z., Lazarević-Pasti T.D., Bondžšić A.M., Vasić V.M. (2013). Acetylcholinesterase inhibitors: Pharmacology and toxicology. Curr. Neuropharmacol..

[100] Sánchez-Guerra M., Pérez-Herrera N., Quintanilla-Vega B. (2011). Organophosphorous pesticides research in Mexico: Epidemiological and experimental approaches. Toxicol. Mech. Methods.

[101] Trueblood A.B., Ross J.A., Shipp E.M., McDonald T.J. (2019). Feasibility of Portable Fingerstick Cholinesterase Testing in Adoles-cents in South Texas. J. Prim. Care Community Health.

[102] Martínez-Valenzuela M.C., Waliszewski S., Gómez-Arroyo S., Villalobos-Pietrini R., Calderón-Vázquez C., Ortega-Martínez D., Meza E., Caba M. (2017). Comparison of organochlorine pesticide levels between human blood serum and adipose tissue. Rev. Int. Contam. Ambient..

[103] Everett C.J., Thompson O.M., Dismuke C.E. (2017). Exposure to DDT and diabetic nephropathy among Mexican Americans in the 1999-2004. National Health and Nutrition Examination Survey. Environ. Pollut..

[104] Flores-Ramírez R., Pérez-Vázquez F.J., Rodríguez-Aguilar M., Medellín-Garibay S.E., Van Brussel E., Cubillas-Tejeda A.C., Carrizales-Yáñez L., Díaz-Barriga F. (2017). Biomonitoring of persistent organic pollutants (POPs) in child populations living near contaminated sites in Mexico. Sci. Total Environ..

[105] Ochoa-Martinez A.C., Orta-Garcia S.T., Rico-Escobar E.M., Carrizales-Yañez L., Martin Del Campo J.D., Pruneda-Alvarez L.G., Ruiz-Vera T., González-Palomo A.K., Piña-Lopez I.G., Torres-Dosal A. (2016). Exposure Assessment to Environmental Chemicals in Children from Ciudad Juarez, Chihuahua, Mexico. Arch. Environ. Contam. Toxicol..

[106] Watkins D.J., Fortenberry G.Z., Sánchez B.N., Barr D.B., Panuwet P., Schnaas L., Osorio-Valencia E., Solano-González M., Ettinger A.S., Hernández-Ávila M. (2016). Urinary 3-phenoxybenzoic acid (3-PBA) levels among pregnant women in Mexico City: Distribution and relationships with child neurodevelopment. Environ. Res..

[107] O’Rourke M.K., Lizardi P.S., Rogan S.P., Freeman N.C., Aguirre A., Saint C.G. (2000). Pesticide exposure and creatinine varia-tion among young children. J. Exp. Anal. Environ. Epidemiol..

[108] Weppner S., Elgethun K., Lu C., Hebert V., Yost M.G., Fenske R.A. (2006). The Washington aerial spray drift study: Children’s exposure to methamidophos in an agricultural community following fixed-wing aircraft applications. J. Exp. Sci. Environ. Epidemiol..

[109] Lee S.J., Mehler L., Beckman J., Diebolt-Brown B., Prado J., Lackovic M., Waltz J., Mulay P., Schwartz A., Mitchell Y. (2011). Acute pesticide illnesses associated with off-target pesticide drift from agricultural applications: 11 states, 1998–2006. Environ. Health Perspect..

[110] Wong F., Alegria H.A., Bidleman T.F., Alvarado V., Angeles F., Galarza A.A., Bandala E.R., Hinojosa Ide L., Estrada I.G., Reyes G.G. (2009). Passive air sampling of organochlorine pesticides in Mexico. Environ. Sci. Technol..

[111] Ramírez-López J.A., Galindo-Reyes J.G. (2014). Los Agroquímicos en la Region sur del Estado de Sinaloa.

[112] Caba M., Meza E., Waliszewski S.M., Martínez-Valenzuela C. (2015). Inverse correlation among organochlorine pesticide levels to total lipid serum contents: A preliminary study in Veracruz, México. Environ. Monit. Assess..

[113] Sexton K., Salinas J.J. (2014). Concurrent fetal exposure to multiple environmental chemicals along the U.S.-Mexico border: An exploratory study in Brownsville, Texas. Int. J. Environ. Res. Public Health.

[114] Mackness M.I., Arrol S., Durrington P.N. (1991). Paraoxonase prevents accumulation of lipoperoxides in low-density lipoprotein. FEBS Lett..

[115] Costa L.G., Cole T.B., Furlong C.E. (2003). Polymorphisms of paraoxonase (PON1) and their significance in clinical toxicology of organophosphates. J. Toxicol. Clin. Toxicol..

[116] Costa L.G., Giordano G., Cole T.B., Marsillach J., Furlong C.E. (2013). Paraoxonase 1 (PON1) as a genetic determinant of suscepti-bility to organophosphate toxicity. Toxicology.

[117] Fortenberry G.Z., Meeker J.D., Sánchez B.N., Bellinger D., Peterson K., Schnaas L., Solano-González M., Ettinger A.S., Hernandez-Avila M., Hu H. (2014). Paraoxonase I polymorphisms and attention/hyperactivity in school-age children from Mexico City, Mexico. Environ. Res..

[118] García-Olmo D.C., Domínguez C., García-Arranz M., Anker P., Stroun M., García-Verdugo J.M., García-Olmo D. (2010). Cell-free nucleic acids circulating in the plasma of colorectal cancer patients induce the oncogenic transformation of susceptible cultured cells. Cancer Res..

[119] Valencia-Quintana R. (2018). Biomarcadores de Daño Genético y Riesgos a la Salud por el Empleo de Plaguicidas en la Producción de Ali-mentos; FORDECYT-PRONACES, FOINS 2016-01 3203. XVII Congreso Internacional y XXIII Congreso Nacional de Ciencias Ambientales.

[120] Jaga K., Dharmani C. (2005). Epidemiology of pesticide exposure and cancer: A review. Rev. Eviron. Health.

[121] Kelada S.N., Eaton D.L., Wang S.S., Rothman N.R., Khoury M. (2003). The role of genetic polymorphisms in environmental health. Environ. Health Perspect..

[122] Aiassa D., Mañas F., Bosch B., Gentile N., Bernard N., Gorla N. (2012). Biomarcadores de daño genético en poblaciones humanas expuestas a plaguicidas. Acta Biol. Colomb..

[123] Bonassi S., Znaor A., Ceppi M., Lando C., Chang W.P., Holland N., Kirsch-Volders M., Zeiger E., Ban S., Barale R. (2007). An increased micronucleus frequency in peripheral blood lymphocytes predicts the risk of cancer in humans. Carcinogenesis.

[124] Tolbert P.E., Shy C.M., Allen J.W. (1992). Micronuclei and other nuclear anomalies in buccal smears: Methods development. Mutat. Res..

[125] Holland N., Bolognesi C., Kirsch-Volders M., Bonassi S., Zeiger E., Knasmueller S., Fenech M. (2008). The micronucleus assay in human buccal cells as a tool for biomonitoring DNA damage: The HUMN project perspective on current status and knowledge gaps. Mutat. Res..

[126] Bonassi S., Coskun E., Ceppi M., Lando C., Bolognesi C., Burgaz S., Holland N., Kirsh-Volders M., Knasmueller S., Zeiger E. (2011). The HUman MicroNucleus project on eXfoLiated buccal cells (HUMN(XL)): The role of life-style, host factors, occupational exposures, health status, and assay protocol. Mutat. Res..

[127] Ceppi M., Biasotti B., Fenech M., Bonassi S. (2010). Human population studies with the exfoliated buccal micronucleus assay: Statistical and epidemiological issues. Mutat. Res..

[128] Ortega-Martínez L.D., Martínez-Valenzuela C., Huerta de la Peña A., Ocampo-Mendoza J., Sandoval-Castro E., Jaramil-lo-Villanueva J.L. (2014). Uso y manejo de plaguicidas en invernaderos de la región norte del estado de Puebla, México. Acta Univ..

[129] Şardaş S. (2005). Genotoxicity tests and their use in occupational toxicology as biomarkers. Indoor Built Envion..

[130] Collins A.R., Azqueta A. (2012). DNA repair as a biomarker in human biomonitoring studies; further applications of the comet assay. Mutat. Res..

[131] Møller P. (2018). The comet assay: Ready for 30 more years. Mutagenesis.

[132] Møller P., Azqueta A., Boutet-Robinet E., Koppen G., Bonassi S., Milić M., Gajski G., Costa S., Teixeira J.P., Costa Pereira C. (2020). Minimum Information for Reporting on the Comet Assay (MIRCA): Recommendations for describing comet as-say procedures and results. Nat. Protoc..

[133] Dusinska M., Collins A.R. (2008). The comet assay in human biomonitoring: Gene-environment interactions. Mutagenesis.

[134] Milić M., Ceppi M., Bruzzone M., Azqueta A., Brunborg G., Godschalk R., Koppen G., Langie S., Møller P., Teixeira J.P. (2021). The hCOMET project: International database comparison of results with the comet assay in human biomonitoring. Baseline frequency of DNA damage and effect of main confounders. Mutat. Res. Rev. Mutat. Res..

[135] Neri M., Milazzo D., Ugolini D., Milic M., Campolongo A., Pasqualetti P., Bonassi S. (2015). Worldwide interest in the comet assay: A bibliometric study. Mutagenesis.

[136] Sánchez-Alarcón J., Milić M., Gómez-Arroyo S., Montiel-González J.M.R., Valencia-Quintana R., Larramendy M.L., Soloneski S. (2016). Assessment of DNA Damage by Comet Assay in Buccal Epithelial Cells: Problems, Achievement, Perspectives. Environmental Health Risk—Hazardous Factors to Living Species.

[137] Silvério A.C.P., Machado S.C., Azevedo L., Nogueira D.A., de Castro Graciano M.M., Simoes J.S., Nachado Viana A.L., Martins I. (2017). Assessment of exposure to pesticides in rural workers in southern of Minas Gerais, Brazil. Environ. Toxicol. Pharmacol..

